# Inhibitors of BRD4 Protein from a Marine-Derived Fungus *Alternaria* sp. NH-F6

**DOI:** 10.3390/md15030076

**Published:** 2017-03-16

**Authors:** Hui Ding, Dashan Zhang, Biao Zhou, Zhongjun Ma

**Affiliations:** Institute of Marine Biology, Ocean College, Zhejiang University, Zhoushan Campus, No. 1 Zheda Road, Zhoushan 316021, China; snnu_dh@163.com (H.D.); 21634129@zju.edu.cn (D.Z.); zhoubiaotd@sina.com (B.Z.)

**Keywords:** marine-derived fungus, *Alternaria* sp. NH-F6, secondary metabolites

## Abstract

Bromodomains (BRD) are readers of the epigenetic code that regulate gene transcription through their recognition of acetyl-lysine modified histone tails. Recently, bromodomain-containing proteins such as BRD4 have been demonstrated to be druggable through the discovery of potent inhibitors. These protein–protein interaction inhibitors have the potential to modulate multiple diseases by their profound anti-inflammatory and antiproliferative effects. In order to explore new BRD4 inhibitors as well as lead compounds for the development of new drugs, the secondary metabolites of *Alternaria* sp. NH-F6, a fungus isolated from deep-sea sediment samples, were analyzed systematically. Five new compounds including two new perylenequinones (**1**–**2**), one new alternaric acid (**3**), 2-(*N*-vinylacetamide)-4-hydroxymethyl-3-ene-butyrolactone (**4**), one new cerebroside (**5**), together with 19 known compounds (**6**–**24**) were isolated from the ethyl acetate extracts of this strain. Their structures were elucidated using nuclear magnetic resonance (NMR) and high resolution electrospray ionization mass spectrometry (HR-ESI-MS) analyses. Finally, all these compounds were evaluated for their inhibitory activity against BRD4 protein, and compound **2** exhibited a potent inhibition rate of 88.1% at a concentration of 10 µM. This research provides a new BRD4 inhibitor which may possess potential antitumoral, antiviral, or anti-inflammatory pharmaceutical values.

## 1. Introduction

Marine-derived fungi have proved to be a major source of natural products due to their complex genetic background and chemodiversity [1,2]. Marine sediments represent an unexplored resource for the isolation of fungal strains [3]. Their various secondary metabolites usually have uncommon structures and potent biological activities that might possess substantial pharmaceutical values [4].

In the field of drug discovery, epigenetic targets are attracting the attention of more and more researchers. Proteins of this kind of targets are mainly classified into readers, writers, and erasers of marks on histones, DNA, or other nuclear proteins [5]. These posttranslational marks regulate gene expression after making complex combinations and have been demonstrated as “histone code” [6]. Bromodomains represent one of the readers of these marks. A bromodomain is an approximately 110 amino acid protein domain that regulates the structure of chromatin, and thereby gene expression, through specially recognizing the acetylated-lysine state of histone tails [7]. Bromodomain-containing proteins have been associated with a number of diseases, including cancers, human immunodeficiency virus (HIV) infection, and neurological disorders [8]. Recent studies show that small molecule modulation of the acetyl-lysine binding activity of BRD proteins such as the bromodomain and extra-terminal domain (BET) family protein BRD4 dictates gene transcription outcome in disease models such as lymphoma, ischemia, and HIV-associated kidney disease, indicating BRD4 protein as an attractive drug target for pathologies including cancer and inflammation [9].

In order to explore new BRD4 inhibitors and lead compounds for the development of new drugs from marine natural products, the secondary metabolites of *Alternaria* sp. NH-F6, a fungus isolated from deep-sea sediment samples collected from the South China Sea, were analyzed systematically. As a result, five new compounds including two new perylenequinones (**1**–**2**), one new alternaric acid (**3**), 2-(*N*-vinylacetamide)-4-hydroxymethyl-3-ene-butyrolactone (**4**), one new cerebroside (**5**), together with 19 known compounds (**6**–**24**) were isolated from the ethyl acetate extracts of the strain’s fermentation products. All the isolated compounds were evaluated for their inhibitory activity against BRD4 protein. Among them, compound **2** exhibited a potent inhibition rate of 88.1% at a concentration of 10 µM, which suggested that compound **2** might possess potential antitumoral, antiviral, or anti-inflammatory pharmaceutical values. This paper describes the isolation, identification, and inhibitory activity of compounds **1**–**24** against BRD4 protein and provides a new BRD4 inhibitor which may make a contribution to drug discovery.

## 2. Results

### 2.1. Identification and Structure Elucidation

**Figure 1:**
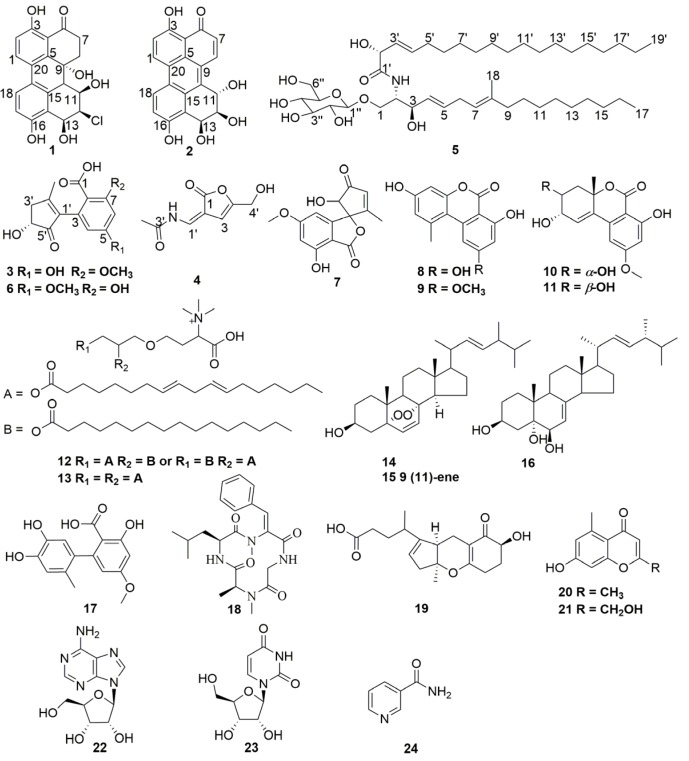
Structures of compounds **1**–**24**.

Compounds **1**–**24** (Figure 1) were obtained by the isolation procedures as described in the materials and methods section.

Compound **1** was obtained as a red amorphous powder, HR-ESI-MS in negative ion mode showed ion *m*/*z* 387.0641 [M − H]^−^. The 3:1 isotopic peak confirmed that there was a chlorine atom in the structure. From the NMR data (Table 1), the number of carbon and hydrogen atoms were easily observed. ChemDraw software was used to calculate the sum of all atoms’ atomic mass (calculation method see Table S2, Supplementary Data). The molecular formula was assigned as C_20_H_17_ClO_6_, with 12 degrees of unsaturation, calculated for C_20_H_16_ClO_6_ *m*/*z* 387.0641 [M − H]^−^, which was the same as the result of HR-ESI-MS.

**Figure 2:**
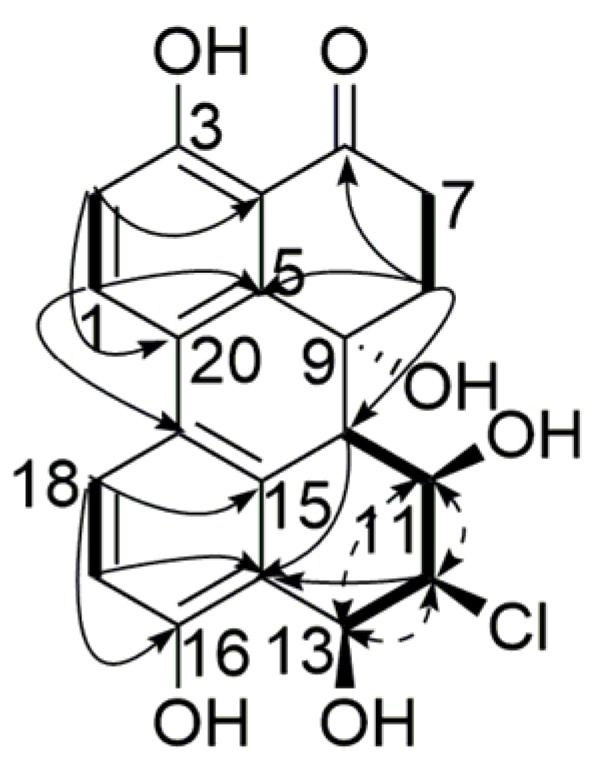
Key HMBC (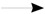), ^1^H-^1^H COSY (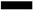) and NOESY (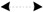) correlations of compound **1**.

The ^1^H NMR spectrum displayed two sets of *ortho*-coupled aromatic protons (δ_H_ 7.97, H-1, 6.98, H-2, 1H each, d, 8.8 Hz), (δ_H_ 6.80, H-17, 7.49, H-18, 1H each, d, 8.5 Hz), five exchangeable hydrogen signals (δ_H_ 12.68, 3-OH, 5.01, 9-OH, 5.72, 13-OH, 9.89, 16-OH, 1H each, s), (δ_H_ 5.12, 11-OH, 1H, d, 5.0 Hz) along with several *sp*^3^ methylene or methine groups. The ^13^C NMR (Table 1) and heteronuclear singular quantum correlation (HSQC) spectra showed resonances for one carbonyl carbon, four aromatic methine carbons, eight aromatic quaternary carbons, four aliphatic methine carbons, two aliphatic methylene carbons, and one aliphatic quaternary carbon, which characterized **1** as a tetrahydroperylenone [10]. The NMR data were very similar to those of dihydroalterperylenol in rings A and B, as well as 8β-Chloro-3, 6aα,7β,9β,10-pentahydroxy-9,8,7,6a-tetrahydroperylen-4(6a*H*)-one in rings D and E compared with the literature [11]. The *p*-dihydroxy diphenyl moiety of rings A and C was evidenced by the long range correlations of H-1/C-3, C-5, C-19, H-2/C-4, C-20, 3-OH/C-2, C-4, H-17/C-14, C-16, C-19, H-18/C-15, C-16, C-20 in heteronuclear multiple bond correlation (HMBC) spectrum. HMBC correlations of H-7/C-6, H-7/C-9, H-8/C-5, C-6, C-9, C-10 together with correlations of H-7/H-8 in homonuclear chemical shift correlation spectroscopy (^1^H-^1^H COSY) confirmed the structure of ring B. The 11,13-dihydroxy substitution in ring E was assigned by ^1^H-^1^H COSY correlations of H-10/H-11, H-11/11-OH, H-11/H-12, H-12/H-13, and H-13/13-OH. The remaining chloride was located only at C-12. The relative configuration of the substitutions in ring E was identified by nuclear overhauser effect spectroscopy (NOESY) and the coupling constants. NOESY spectrum showed correlations of H-11 with H-12, H-12 with H-13, and H-11 with H-13. The coupling constant of *J*_10,11_ = 9.3 Hz, suggested that H-10/H-11 was *trans*-oriented with typical axial-axial interaction [11]. The small coupling constants of *J*_11,12_ = 2.9 Hz and *J*_12,13_ = 3.0 Hz were indicative of *cis* orientation of H-11/H-12/H-13 [11]. Consequently, the structure of compound **1** was determined to be 12β-Chloro-3,9α,11β,13β,16-pentahydroxy-8,9,10,11,12,13-hexahydro-6(7*H*)-one (Figure 2).

**Figure 3:**
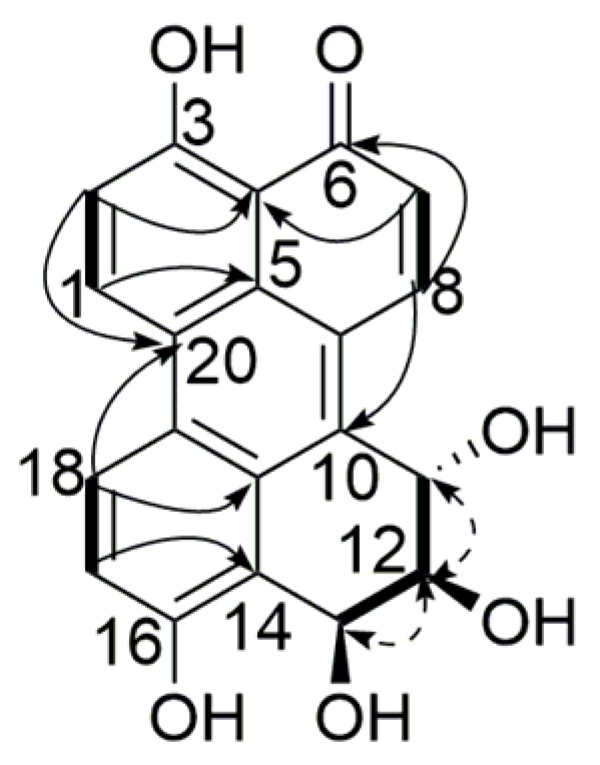
Key HMBC (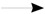), ^1^H-^1^H COSY (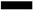) and NOESY (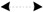) correlations of compound **2**.

Compound **2** was obtained as a red amorphous powder, and its molecular formula was assigned as C_20_H_14_O_6_, with 14 degrees of unsaturation, from its HR-ESI-MS (*m*/*z* 351.0862 [M + H]^+^, calcd. for C_20_H_15_O_6_ 351.0863) and NMR data (Table 1). The ^1^H NMR spectrum displayed three sets of *ortho*-coupled aromatic protons (δ_H_ 9.21, H-1, 7.47, H-2, 1H each, d, 9.2 Hz), (δ_H_ 7.04, H-7, 8.79, H-8, 1H each, d, 10.0 Hz), (δ_H_ 7.43, H-17, 8.81, H-18, 1H each, d, 9.2 Hz), four exchangeable hydrogen signals (δ_H_ 15.23, 3-OH, 10.54, 16-OH, 1H each, s), (δ_H_ 5.51, 12-OH, 1H, d, 5.4 Hz), (δ_H_ 5.72, 13-OH, 1H, d, 5.3 Hz) along with three *sp*^3^ methine groups (δ_H_ 5.33, H-11, 1H, d, 8.5 Hz), (δ_H_ 3.92, H-12, 1H, ddd, 8.5, 5.4, 3.3 Hz), (δ_H_ 5.65, H-13, 1H, dd, 5.3, 3.3 Hz). The ^13^C NMR (Table 1) and HSQC spectra showed resonances for 1 carbonyl carbon, 6 aromatic methine carbons, 10 aromatic quaternary carbons, and 3 aliphatic methine carbons. These signals together indicated that compound **2** had similar structure to compound **1**. HMBC correlations of H-7/C-4, C-9 and H-8/C-5, C-6, C-10 suggested that the additional aromatic methine and quaternary carbons were located in ring B and ring C, respectively. ^1^H-^1^H COSY correlations of H-11/H-12, H-12/12-OH, and H-12/H-13 confirmed the 11,12,13-trihydroxy substitution in ring E. The relative configuration of the substitutions in ring E was identified by the same method described above. The absent correlation signal of H-11/H-13 in the NOESY spectrum, together with the coupling constants of *J*_11,12_ = 8.5 Hz and *J*_12,13_ = 3.3 Hz suggested that H-11/H-12 was *trans*-oriented while H-12/H-13 was *cis*-oriented. Consequently, the structure of compound **2** was determined to be 3,11α,12β,13β,16-pentahydroxy-11,12-dihydroperylen-6(13*H*)-one (Figure 3).

**Figure 4:**
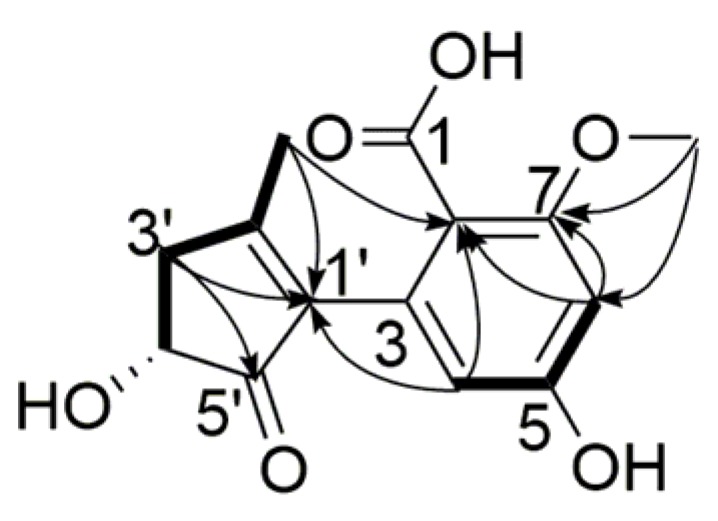
Key HMBC (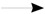) and ^1^H-^1^H COSY (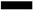) correlations of compound **3**.

Compound **3** was obtained as a viscous, yellow oil, and its molecular formula was assigned as C_14_H_14_O_6_, with eight degrees of unsaturation, from its HR-ESI-MS (*m*/*z* 277.0724 [M − H]^−^, calcd. for C_14_H_13_O_6_ 277.0718) and NMR data (Table 2). The ^1^H NMR spectrum displayed two aromatic protons (δ_H_ 6.35, H-6, 5.95, H-4, 1H each, s), one methoxyl group (δ_H_ 3.74, 7-OCH_3_, 3H, s), one methyl group (δ_H_ 1.90, 2′-CH_3_, 3H, s), one methine group (δ_H_ 4.21, H-4′, 1H, d, 6.5 Hz), and one methylene group (δ_H_ 2.90, H-3′a, 1H, dd, 17.1, 6.5 Hz), (δ_H_ 2.41, H-3′b, 1H, d, 17.1 Hz). The ^13^C NMR spectrum exhibited a total of 14 carbon resonances including 10 *sp*^2^ carbon atoms and 4 *sp*^3^ carbon atoms (Table 2). The data displayed similar signals to alternarienonic acid (**6**) reported in the literature [12] except the ^13^C chemical shifts at C-1, C-5, and C-7. This indicated that the substituent groups at C-1, C-5, and C-7 were different from those of compound **6**. ^1^H-^1^H COSY correlation of H-4/H-6 along with HMBC correlations of H-4/C-1′, C-6, C-7, H-6/C-2, C-4, C-7, 7-OCH_3_/C-1, C-6, C-7, 2′-CH_3_/C-2, C-4 indicated that the carboxyl, methoxyl, and hydroxyl groups were located at C-1, C-7, and C-5, respectively. The NMR signals at C-4′ (δ_H_ 4.21, δ_C_ 71.4) were exactly similar to those of compound **6**, so the configuration of C-4′ was determined to be *R*. Consequently, the structure of compound **3** was assigned as shown, and this compound has been named alternarienonic acid B (Figure 4).

**Figure 5:**
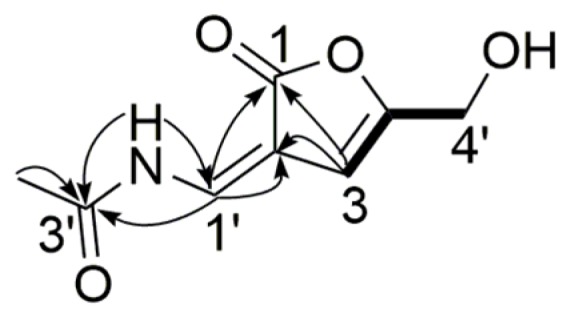
Key HMBC (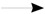) and ^1^H-^1^H COSY (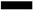) correlations of compound **4**.

Compound **4** was obtained as a white powder, and its molecular formula was assigned as C_8_H_9_NO_4_, with five degrees of unsaturation, from its HR-ESI-MS (*m*/*z* 206.0426 [M + Na]^+^, calcd. for C_8_H_9_NO_4_Na 206.0424) and NMR data (Table 3). The ^1^H and ^13^C NMR spectra displayed two *sp*^2^ methine groups (δ_H_ 9.07, H-1′, 1H, s, δ_C_ 144.3, C-1′), (δ_H_ 6.40, H-3, 1H, s, δ_C_ 109.5, C-3), one methylene group (δ_H_ 4.32, H-4′, 2H, s, *δ*_C_ 59.4, C-4′), one methyl group (δ_H_ 2.10, 3′-CH_3_, 3H, s, δ_C_ 23.3, 3′-CH_3_), and two exchangeable protons (δ_H_ 9.25, 2′-NH, 1H, s), (δ_H_ 5.73, 4′-OH, 1H, s), which were assigned by HSQC spectrum. The hydroxymethyl substituted 3-ene-butyrolactone moiety was evidenced by ^1^H-^1^H COSY correlation of H-3/H-4′ and HMBC correlations of H-3/C-2, C-1, C-4, C-4′, H-4′/C-4. The singlet of H-3 and H-4′ in ^1^H NMR spectrum confirmed that they were only located at position 3 and 4′, respectively. Correlations of 3′-CH_3_/C-3′, 2′-NH/C-3′, C-1′ and H-1′/C-3′, C-2, C-1 indicated that a *N*-vinylacetamide group was connected to the 3-ene-butyrolactone moiety. Consequently, the structure of compound **4** was determined to be 2-(*N*-vinylacetamide)-4-hydroxymethyl-3-ene-butyrolactone (Figure 5).

**Figure 6:**
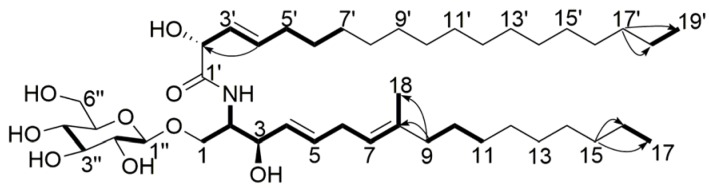
Key HMBC (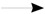) and ^1^H-^1^H COSY (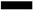) correlations of compound **5**.

Compound **5** was obtained as a white powder, and its molecular formula was assigned as C_43_H_79_NO_9_, with five degrees of unsaturation, from its HR-ESI-MS (*m*/*z* 754.5827 [M + H]^+^, calcd. for C_43_H_80_NO_9_ 754.5828) and NMR data (Table 4). The ^1^H NMR spectrum displayed two sets of *ortho*-coupled protons (δ_H_ 5.45, H-4, 1H, dd, 14.8, 6.0 Hz), (δ_H_ 5.72, H-5, 1H, d, 14.8 Hz), (δ_H_ 5.49, H-3′, 1H, dd, 15.8, 4.2 Hz), (δ_H_ 5.85, H-4′, 1H, dt, 15.8, 5.8 Hz), one *sp^2^* proton (δ_H_ 5.08, H-7, 1H, t, 6.0 Hz), three methyl groups (δ_H_ 0.89, H-17, H-19′; 3H each, t, 6.8 Hz), (δ_H_ 1.57, H-18, 3H, s), one exchangeable proton (δ_H_ 7.39, NH, 1H, s), and several oxidized *sp^3^* methine or methylene groups. The ^13^C NMR spectrum showed seven *sp^2^* carbon atoms and nine oxidized *sp^3^* carbon atoms. The overlap signals (δ_H_ 1.26, 36H, δ_C_ 29.4–32.1) indicated the existence of long aliphatic carbon chains in the structure. The NMR data were exactly similar to those of chrysogeside D reported in the literature [13], which suggested that they were analogs. The difference between the two compounds was that compound **5** had only one methylene group between C-5 and C-7. Correlations of H-5/H-4, H-5/H-6, H-6/H-7, H-7/H-18, H-9/H-10 in ^1^H-^1^H COSY spectrum, together with correlations of H-7/C-6, H-9/C-6, C-8, C-10, C-18, H-18/C-6, C-8, C-9 in HMBC spectrum confirmed that there was only one methylene group between C-5 and C-7. The methanolysis products of compound **5** were further analyzed by HR-ESI-MS in order to determine the lengths of the aliphatic carbon chains. When the fragments *m*/*z* 217.0678 [M + Na]^+^ (calcd. for C_7_H_14_O_6_Na 217.0683), and the negative mode *m*/*z* 229.0488 [M + Cl]^−^ (calcd. for C_7_H_14_ClO_6_ 229.0484), along with the ^13^C NMR data were compared with those of chrysogeside D, it indicated that compound **5** had the same methyl d-glucopyranoside moiety as chrysogeside D. The NMR signals of the anomeric proton and carbon at δ_H_ 4.37/δ_C_ 103.1 suggested the β-configuration of the glucoside [14]. Besides, the same C-19 fatty acid fragment *m*/*z* 335.2558 [M + Na]^+^ (calcd. for C_19_H_36_O_3_Na 335.2557) and its methyl ester *m*/*z* 349.2713 [M + Na]^+^ (calcd. for C_20_H_38_O_3_Na 349.2713) were also detected in the HR-ESI-MS spectrum. The observation of a 15.8 Hz coupling between H-3′ and H-4′ allowed assignment of the *E* configuration to this double bond. Furthermore, a C-18 unsaturated fatty acid fragment *m*/*z* 298.2738 [M + H]^+^ (calcd. for C_18_H_36_NO_2_ 298.2741) and its methyl ester *m*/*z* 312.2896 [M + H]^+^ (calcd. for C_19_H_38_NO_2_ 312.2897) were detected. The *E* configuration of the C-4, C-5 double bond was assigned on the basis of a large coupling (15.8 Hz) between H-4 and H-5. The ^13^C chemical shifts at C-1, C-2, C-3, C-1′, and C-2′ were very similar to the related cerebrosides reported in the literature [15], so the absolute configuration of C-2, C-3, and C-2′ was determined to be *S*, *R*, *R*, respectively. Consequently, the structure of compound **5** was assigned as (2*R*,3*E*)-2-hydroxy-*N*-[(2*S*,3*R*,4*E*,7*E*)-1-β-d-glucopyranosyloxy-3-hydroxy-8-methylheptadec-4,7-dien-2-yl]nonadec-3-enamide (Figure 6), and this compound has been named chrysogeside F.

Compound **6** was identified as alternarienonic acid by comparison with data in reference [12]. Compound **7** was identified as talaroflavone by comparison with data in reference [16]. Compound **8** was identified as alternariol by comparison with data in reference [12]. Compound **9** was identified as alternariol 5-*O*-methyl ether by comparison with data in reference [17]. Compound **10** was identified as 4′-epialtenuene by comparison with data in reference [12]. Compound **11** was identified as altenuene by comparison with data in reference [18]. Compound **12** was identified as 1(2)-linolyl-2(1)-palmityl-glycero-*O*-4′-(*N*,*N*,*N*-trimethyl) homoserine by comparison with data in reference [19]. Compound **13** was identified as 1,2-dilinolylglycero-*O*-4′-(*N*,*N*,*N*-trimethyl) homoserine by comparison with data in reference [19]. Compound **14** was identified as 5,8-epidioxy-5α,8α-ergosta-6, 22*E*-dien-3β-ol by comparison with data in reference [20]. Compound **15** was identified as 5,8-epidioxy-5α,8α-ergosta-6,9,22*E*-dien-3β-ol by comparison with data in reference [20]. Compound **16** was identified as (22*E*,24*R*)-24-methyl-5α-cholesta-7,22-diene-3β,5,6β-triol by comparison with data in reference [21]. Compound **17** was identified as altenusin by comparison with data in reference [22]. Compound **18** was identified as tentoxin by comparison with data in reference [23]. Compound **19** was identified as tricycloalternarene A by comparison with data in reference [24]. Compound **20** was identified as 2,5-dimethyl-7-hydroxychromone by comparison with data in reference [25]. Compound **21** was identified as 7-hydroxy-2-hydroxymethyl-5-methyl-4*H*-chromen-4-one by comparison with data in reference [26]. Compound **22** was identified as β-adenosine by comparison with data in reference [27]. Compound **23** was identified as uridine by comparison with data in reference [28]. Compound **24** was identified as nicotinamide by comparison with data in reference [29].

### 2.2. Inhibitory Activity against BRD4 Protein

The inhibitory activity of compounds **1**–**24** against BRD4 protein was evaluated using the BRD4 bromodomain 1 (BD1) inhibitor screening assay kit and the positive control was JQ1 (Table 5). Among these compounds, compound **2** exhibited a potent inhibition rate of 88.1%. Compound **1** showed a moderate inhibition rate of 57.7%. The inhibition rates of the other compounds were all below 35.0% when tested at a concentration of 10 µM.

## 3. Discussion

As epigenetic readers of the histone code, BET family proteins (including BRD2, BRD3, BRD4, and BRDT) play an important role in a number of human diseases. Among them, BRD4 is the most extensively studied member. It contains two highly conserved N-terminal bromodomains (BD1 and BD2) that recognize acetylated lysine residues, an extraterminal domain and a C-terminal domain which has little sequence homology to other BET family members [30]. BD1 and BD2 interact with acetylated chromatin and non-histone proteins to regulate transcription, DNA replication, cell cycle progression, and other cellular activities [31]. The C-terminal domain interact with the transcription elongation factor P-TEFb as well as RNA polymerase II and has been implicated in promoting gene transcription [31]. As literature reports, disrupting the protein-protein interactions between BRD4 and acetyl-lysine can effectively block cell proliferation in cancer [32,33], cytokine production in acute inflammation [34], as well as alleviate HIV-1 latency [35]. BRD4 is thus considered as a promising therapeutic target for multiple diseases and targeting BRD4 has attracted significant interest of researchers in drug discovery. Although a number of divers BRD4 inhibitors have been reported, very few of them exhibit excellent selectivity among BET family members or sub-bromodomains [36]. Thus, more potent and specific BRD4 inhibitors are still urgently needed.

In the course of our search for new BRD4 inhibitors as well as lead compounds from marine-derived fungi, a strain of *Alternaria* sp. NH-F6 was isolated from deep-sea sediment samples collected from South China Sea. The ethyl acetate extracts of the fermentation products yielded two new perylenequinones (**1**–**2**), one new alternaric acid (**3**), 2-(*N*-vinylacetamide)-4-hydroxymethyl-3-ene-butyrolactone (**4**), one new cerebroside (**5**), together with 19 known compounds including alternarienonic acid (**6**), talaroflavone (**7**), alternariol (**8**), alternariol 5-*O*-methyl ether (**9**), 4′-epialtenuene (**10**), altenuene (**11**), diacylglycerotrimethyl homoserine lipids (**12**–**13**), 5,8-epidioxy-5α,8α-ergosta-6,22*E*-dien-3β-ol (**14**), 5,8-epidioxy-5α,8α-ergosta-6,9,22*E*-dien-3β-ol (**15**), (22*E*,24*R*)-24-methyl-5α-cholesta-7,22-diene-3β,5,6β-triol (**16**), altenusin (**17**), tentoxin (**18**), tricycloalternarene A (**19**), 2,5-dimethyl-7-hydroxychromone (**20**), 7-hydroxy-2-hydroxymethyl-5-methyl-4*H*-chromen-4-one (**21**), β-adenosine (**22**), uridine (**23**), and nicotinamide (**24**). These metabolites were mainly classified into quinones, glycerides, steroids, pyranones, nitrogen-containing compounds, terpenoids, and phenolics, and the structural types were almost the same as those reported in *Alternaria* species [37]. Altertoxins have been reported in the literature from several *Alternaria* species [37]. These compounds have similar structures to compounds **1** and **2** and they usually have epoxides in the structures. The epoxides in the altertoxins could be opened under mild conditions to form compounds like **1** and **2**.

BRD4 inhibitor screening assay showed that compound **2** was a potent inhibitor with an inhibition rate of 88.1% at a concentration of 10 µM. Compound **1** showed a moderate inhibition rate of 57.7% when tested at the same concentration. This indicated that they might also possess significant antitumor, antiviral, or anti-inflammatory activities. The discovery of new BRD4 inhibitors may provide reference for drug design and make a contribution to the treatment of cancer or other diseases. Currently, more than a dozen of BRD4 inhibitors have progressed into human clinical trials for the treatment of cancer or other diseases [38]. These molecules usually have a unique head moiety that can form critical hydrogen bonds with residues of the BRD4 binding pocket. Among the hydrogen bond, they often contain a small hydrophobic group such as a methyl group [38]. Since compounds **1** and **2** are structurally different from these molecules, the exact mechanism of its inhibitory effect against BRD4 protein will be explored in our future research.

## 4. Experimental Section

### 4.1. General

HR-ESI-MS spectra were obtained using Agilent-6230Q-TOF mass spectrometer (Agilent Technologies China Co., Ltd., Shanghai, China). Ultra-high purity helium was used as the collision gas and high-purity nitrogen as the nebulizing gas. 1D- and 2D-NMR spectra were recorded on Bruker Ascend 600 and 400 MHz spectrometers (Bruker Technologies Beijing Co., Ltd., Beijing, China) using tetramethylsilane as the internal standard. Infrared ray (IR) spectra were recorded on Bruker Vector 22 (Bruker Technologies Beijing Co., Ltd., Beijing, China) and optical rotations were measured by JASCO P1010 (JASCO China Co., Ltd., Shanghai, China). High performance liquid chromatography (HPLC) analyses were performed on Shimadzu LC-20 AT prominence liquid chromatograph (Shimadzu China Co., Ltd., Shanghai, China). Preparative HPLC using a LC-3000 system with photodiode array detector purchased from Beijing Chuangxintongheng Science & Technology Co., Ltd. (Beijing, China), was used to purify compounds isolated from atmospheric silica gel column chromatography. A Pursuit XRs 10 C18 column (250 × 21.2 mm) was used for preparative HPLC. Preparative medium performance liquid chromatography (MPLC) was carried out on an octadecylsilyl C_18_ column (25 mm × 500 mm). Time-resolved fluorescence resonance energy transfer (TR-FRET) signals were measured by multifunctional microplate reader SpectraMax M5. BRD4 (BD1) inhibitor screening assay kit was purchased from Cisbio Bioassays (Codolet, France). Silica gel (100–200 mesh) was purchased from Qingdao Haiyang Chemical Group Co., Ltd., (Qingdao, China). Sephadex LH-20 was purchased from GE Healthcare (Beijing, China). Dichloromethane and methanol were purchased from Tianjin Damao Chemical Reagents Co., Ltd. (Tianjin, China). Deionized water was prepared using a Milli-Q system (Millipore, Bedford, MA, USA).

### 4.2. Fungus Material

*Alternaria* sp. NH-F6 was isolated by the first author using the standard agar plate dilution method from the marine sediments collected from Sansha City (16°83′ N, 112°48′ E), South China Sea, in October 2015. Sediments were sampled at a depth of 3927 m. The fungus was identified by observing the morphological characteristics and analysis of the 26s ribosomal deoxyribose nucleic acid (rDNA) regions (GenBank accession number: KY 378939.1). The strain was deposited in BeNa Culture Collection (BNCC 151307), Beijing, China.

### 4.3. Fermentation and Isolation

*Alternaria* sp. NH-F6 was maintained on potato dextrose agar (PDA) medium at 28 °C for seven days. Spores of the strain were inoculated into 500 mL Erlenmeyer flasks containing 250 mL potato dextrose broth (PDB) medium (200 g potatoes, 20 g glucose, 25 g baysalt, and 1000 mL distilled water) using a sterile inoculation loop and incubated at 28 °C on a rotary shaker at 180 rpm for three days. Large scale solid fermentation was carried out in 200 bottles of 500 mL Erlenmeyer flasks each containing 122 g rice medium (40 g rice, 2 g baysalt, and 80 mL distilled water). Each flask was inoculated with 15.0 mL culture medium and incubated at 28 °C for 15 days.

When the fermentation was finished, the fermentation products were extracted with equal volume of ethyl acetate twice to yield 36.0 g crude extract. This extract was subjected to silica gel column chromatography (6 × 80 cm, 250 g silica gel) and eluted with a gradient of CH_2_Cl_2_–CH_3_OH (1:0, 80:1, 40:1, 20:1, 5:1, 1:1, 0:1, *v*/*v*, each 2.5 L) to give 11 fractions (Fr. A–K).

Fr. B (0.95 g) was subsequently subjected to silica gel column chromatography (3 × 40 cm, 20 g silica gel) and eluted with a gradient of CH_2_Cl_2_–CH_3_OH (150:1, 100:1, 80:1, 40:1, 0:1, *v*/*v*, each 200 mL) to give six fractions (Fr. B-1 to Fr. B-6). Fr. B-4 (33.2 mg) was dissolved in CH_2_Cl_2_ and kept standing for two days to precipitate compound **9** (0.9 mg). Fr. B-5 (347.4 mg) was subjected to silica gel column chromatography (1.5 × 40 cm, 10 g silica gel) and eluted with a gradient of CH_2_Cl_2_–CH_3_OH (100:1, 60:1, 30:1, 15:1, 0:1, *v*/*v*, each 100 mL) to give four fractions (Fr. B-5-1 to Fr. B-5-4). Fr. B-5-2 (52.0 mg) was fractionated by preparative HPLC using 95% CH_3_OH-H_2_O as the mobile phase (flow rate 10 mL/min, λ = 210 nm) to give compound **15** (6.3 mg, *t*_R_ = 27 min) and compound **14** (19.7 mg, *t*_R_ = 33 min). Fr. B-6 (103.5 mg) was subjected to silica gel column chromatography (1.5 × 40 cm, 10 g silica gel) and eluted with a gradient of CH_2_Cl_2_–CH_3_OH (80:1, 40:1, 20:1, 15:1, 0:1, *v*/*v*, each 100 mL) to give four fractions (Fr. B-6-1 to Fr. B-6-4). Fr. B-6-3 (66.4 mg) was purified on preparative HPLC using 45% CH_3_OH-H_2_O as the mobile phase (flow rate 10 mL/min, λ = 210 nm) to give compound **20** (4.7 mg, *t*_R_ = 33 min).

Fr. D (0.6 g) was subjected to silica gel column chromatography (3 × 40 cm, 20 g silica gel) and eluted with a gradient of CH_2_Cl_2_–CH_3_OH (80:1, 50:1, 20:1, 0:1, *v*/*v*, each 200 mL) to give three fractions (Fr. D-1 to Fr. D-3). Fr. D-2 (254.2 mg) was fractionated by preparative MPLC using a 90 min gradient from 10% CH_3_OH-H_2_O to 100% CH_3_OH-H_2_O (flow rate 10 mL/min, λ = 210 nm). Fr. D-2-1 (*t*_R_ = 38–48 min) and Fr. D-2-2 (*t*_R_ = 66.5–73 min) were recycled and dried at 45 °C under vacuum condition, respectively. Fr. D-2-1 (55.6 mg) was subsequently purified on preparative HPLC (28% CH_3_OH-H_2_O, flow rate 10 mL/min, λ = 210 nm) to give compound **7** (5.3 mg, *t*_R_ = 40 min). Fr. D-2-2 (42.8 mg) was also purified on preparative HPLC (48% CH_3_OH-H_2_O, flow rate 10 mL/min, λ = 210 nm) to give compound **18** (15.7 mg, *t*_R_ = 48 min) and compound **19** (7.3 mg, *t*_R_ = 52 min).

Fr. F (1.21 g) was subjected to silica gel column chromatography (3 × 40 cm, 20 g silica gel) and eluted with a gradient of CH_2_Cl_2_–CH_3_OH (50:1, 30:1, 10:1, 5:1, 0:1, *v*/*v*, each 200 mL) to give five fractions (Fr. F-1 to Fr. F-5). Fr. F-3 (124.1 mg) was fractionated by preparative MPLC using a 90 min gradient from 10% CH_3_OH-H_2_O to 100% CH_3_OH-H_2_O (flow rate 10 mL/min, λ = 210 nm). Fr. F-3-1 (*t*_R_ = 19–25 min) and Fr. F-3-2 (*t*_R_ = 55–70 min) were recycled and dried at 45 °C under vacuum condition, respectively. Fr. F-3-1 (12.0 mg) was subsequently purified on preparative HPLC (20% CH_3_OH-H_2_O, flow rate 10 mL/min, λ = 210 nm) to give compound **4** (4.0 mg, *t*_R_ = 32 min). Fr. F-3-2 (73.2 mg) was also purified on preparative HPLC (38% CH_3_OH-H_2_O, flow rate 10 mL/min, λ = 210 nm) to give compound **11** (28.2 mg, *t*_R_ = 74 min) and compound **10** (9.2 mg, *t*_R_ = 81 min). Fr. F-4 (46.0 mg) was purified on Sephadex LH-20 column chromatography (3 × 40 cm, 50 g gel) and eluted with CH_3_OH to give compound **6** (9.0 mg).

Fr. G (1.25 g) was subjected to silica gel column chromatography (3 × 40 cm, 20 g silica gel) and eluted with a gradient of CH_2_Cl_2_–CH_3_OH (60:1, 30:1, 20:1, 0:1, *v*/*v*, each 200 mL) to give five fractions (Fr. G-1 to Fr. G-5). Fr. G-4 (460.0 mg) was fractionated by preparative MPLC using a 90 min gradient from 10% CH_3_OH-H_2_O to 100% CH_3_OH-H_2_O (flow rate 10 mL/min, λ = 210 nm). Fr. G-4-1 (*t*_R_ = 20–25 min), Fr. G-4-2 (*t*_R_ = 30–35 min) and Fr. G-4-3 (*t*_R_ = 37–45 min) were recycled and dried at 45 °C under vacuum condition, respectively. Fr. G-4-1 (4.7 mg) was purified on Sephadex LH-20 column chromatography (1.5 × 40 cm, 25 g gel) and eluted with CH_3_OH to give compound **3** (2.4 mg). Fr. G-4-2 (42.6 mg) was purified on Sephadex LH-20 column chromatography (3 × 40 cm, 50 g gel) and eluted with CH_3_OH to give compound **17** (39.3 mg). Fr. G-4-3 (11.3 mg) was purified on preparative HPLC using 30% CH_3_OH-H_2_O as the mobile phase (flow rate 10 mL/min, λ = 210 nm) to give compound **21** (2.4 mg, *t*_R_ = 59 min). Fr. G-5 (10.2 mg) was also purified on preparative HPLC (55% CH_3_OH-H_2_O, flow rate 10 mL/min, λ = 210 nm) to give compound **8** (1.2 mg, *t*_R_ = 47 min).

Fr. H (2.0 g) was fractionated by silica gel column chromatography (3 × 40 cm, 30 g silica gel) and eluted with a gradient of CH_2_Cl_2_–CH_3_OH (30:1, 20:1, 10:1, 1:1, *v*/*v*, each 300 mL) to give seven fractions (Fr. H-1 to Fr. H-7). Fr. H-3 (161.0 mg) was fractionated by preparative MPLC using a 90 min gradient from 10% CH_3_OH-H_2_O to 100% CH_3_OH-H_2_O (flow rate 10 mL/min, λ = 210 nm) to give compound **16** (3.7 mg, *t*_R_ = 72 min). Fr. H-4 (371.4 mg) was also fractionated by preparative MPLC using a 40 min gradient from 10% CH_3_OH-H_2_O to 100% CH_3_OH-H_2_O (flow rate 10 mL/min, λ = 210 nm). Fr. H-4-1 (*t*_R_ = 9-22 min) and Fr. H-4-2 (*t*_R_ = 37–48 min) were recycled and dried at 45 °C under vacuum condition, respectively. Fr. H-4-1 (63.5 mg) was purified on preparative HPLC using 40% CH_3_OH-H_2_O as the mobile phase (flow rate 10 mL/min, λ = 210 nm) to give compound **1** (4.0 mg, *t*_R_ = 105 min). Fr. H-4-2 (57.3 mg) was subjected to silica gel column chromatography (1 × 20 cm, 3 g silica gel) and eluted with a gradient of CH_2_Cl_2_–CH_3_OH (30:1, 15:1, 8:1, *v*/*v*, each 30 mL) to give compound **2** (2.0 mg). Fr. H-7 (160.1 mg) was purified on preparative HPLC (98% CH_3_OH-H_2_O, flow rate 10 mL/min, λ = 210 nm) to give compound **5** (17.0 mg, *t*_R_ = 48 min).

Fr. I (0.23 g) was subjected to silica gel column chromatography (1.5 × 40 cm, 10 g silica gel) and eluted with a gradient of CH_2_Cl_2_–CH_3_OH (10:1, 5:1, 0:1, *v*/*v*, each 100 mL) to give three fractions (Fr. I-1 to Fr. I-3). Fr. I-3 (9.4 mg) was purified on Sephadex LH-20 column chromatography (1.5 × 40 cm, 25 g gel) and eluted with CH_3_OH to give compound **23** (6.7 mg).

Fr. J (0.64 g) was dissolved in ethyl acetate and kept standing for two days to precipitate compound **22** (9.7 mg).

Fr. K (0.64 g) was subjected to silica gel column chromatography (3 × 40 cm, 20 g silica gel) and eluted with a gradient of CH_2_Cl_2_–CH_3_OH (15:1, 10:1, 5:1, *v*/*v*, each 200 mL) to give four fractions (Fr. K-1 to Fr. K-4). Fr. K-4 (142.6 mg) was fractionated by preparative HPLC using 98% CH_3_OH-H_2_O as the mobile phase (flow rate 10 mL/min, *λ* = 210 nm) to give compound **13** (10.7 mg, *t*_R_ = 62 min) and compound **12** (18.6 mg, *t*_R_ = 78 min).

Fr. L (1.2 g) was subjected to silica gel column chromatography (3 × 40 cm, 20 g silica gel) and eluted with a gradient of CH_2_Cl_2_–CH_3_OH (40:1, 25:1, 5:1, *v*/*v*, each 200 mL) to give five fractions (Fr. L-1 to Fr. L-5). Fr. L-5 (25.2 mg) was purified on preparative HPLC (20% CH_3_OH-H_2_O, flow rate 10 mL/min, λ = 210 nm) to give compound **24** (1.1 mg, *t*_R_ = 15 min).

The flow rate of atmospheric silica gel and Sephadex LH-20 column chromatography described above was regulated by gravity.

### 4.4. Methanolysis of Compound ***5***

Compound **5** (2 mg) was refluxed with 20% HCl–MeOH (5 mL) for 18 h. The products were dried at 42 °C under vacuum conditions. To analyze the products, the sample was directly driven into HR-ESI-MS by an autosampler after dissolving in methanol. The HR-ESI-MS ionization source parameters were set up as follows: the capillary voltage was set up at 4.0 kV. The drying gas temperature was maintained at 325 °C. The nebulizer pressure was set up at 20 psi and the drying gas flow was 5 L/min. The injection volume was 5 μL and the flow rate was set up at 200 μL/min. The sample temperature was maintained at 35 °C.

*12β-Chloro-3,9α,11β,13β,16-pentahydroxy-8,9,10,11,12,13-hexahydro-6(7H)-one* (**1**): red, amorphous powder; ${\left[\alpha \right]}_{\text{D}}^{20}$ +46.0 (*c* 0.10, MeOH); UV (MeOH) λ_max_ 200, 260, 287 nm; IR (film) ν_max_ 3424, 1633, 1026 cm^−1^; ^1^H NMR and ^13^C NMR spectroscopic data, Table 1; HR-ESI-MS [M − H]^−^ *m*/*z* 387.0641 (calcd. for C_20_H_16_ClO_6_, 387.0641).

*3,11α,12β,13β,16-Pentahydroxy-11,12-dihydroperylen-6(13H)-one* (**2**): red, amorphous powder; ${\left[\alpha \right]}_{\text{D}}^{20}$ +9.0 (*c* 0.10, MeOH); UV (MeOH) λ_max_ 199, 226, 257 nm; IR (film) ν_max_ 3442, 1625, 1030 cm^−1^; ^1^H and ^13^C NMR spectroscopic data, Table 1; HR-ESI-MS [M + H]^+^ *m*/*z* 351.0862 (calcd. for C_20_H_15_O_6_, 351.0863).

*Alternarienonic acid B* (**3**): viscous, yellow, oil; ${\left[\alpha \right]}_{\text{D}}^{20}$ +70.0 (c 0.10, MeOH); UV (MeOH) λ_max_ 223, 248, 299 nm; IR (film) ν_max_ 3367, 1701, 1586, cm^−1^; ^1^H and ^13^C NMR spectroscopic data, Table 2; HR-ESI-MS [M − H]^−^ *m*/*z* 277.0724 (calcd. for C_14_H_13_O_6_ 277.0718).

*2-(N-Vinylacetamide)-4-hydroxymethyl-3-ene-butyrolactone* (**4**): white, powder; UV (MeOH) λ_max_ 227, 256 nm; IR (film) ν_max_ 3480, 3361, 1650 cm^−1^; ^1^H and ^13^C NMR spectroscopic data, Table 3; HR-ESI-MS [M + Na]^+^ *m*/*z* 206.0426 (calcd. for C_8_H_9_NO_4_Na 206.0424).

*Chrysogeside F* (**5**): white, powder; ${\left[\alpha \right]}_{\text{D}}^{20}$ −4.0 (*c* 0.10, MeOH); UV (MeOH) λ_max_ 207 nm; IR (film) ν_max_ 3367, 2921, 1071, cm^−1^; ^1^H and ^13^C NMR spectroscopic data, Table 4; HR-ESI-MS [M + H]^+^ *m*/*z* 754.5827 (calcd. for C_43_H_80_NO_9_ 754.5828).

### 4.5. BRD4 Inhibitor Screening Assay

The commercially available assay kit (BRD4 BD1 TR-FRET assay kit) based on time-resolved fluorescence technology was used to evaluate the inhibition rates of BRD4 protein according to the supplier’s protocols. HTRF assays were performed in white 384 Well Shallow Well Standard Height Plates (Nunc™) with a total working volume of 20 μL. Compounds were diluted, with diluent and detection buffer supplied by TR-FRET assay kit (0.8% final DMSO), from a concentration stock of 10 mM in 100% DMSO for the primary screen. All HTRF reagents were reconstituted and diluted according to the supplier’s protocols. For each assay, 5 μL of Biotin-labeled peptide ligand was added to each well at a final concentration of 20.6 nM. 5 μL of His-tagged BRD4-BD1 was then added to each well at a final concentration of 11 nM. Afterwards, 2.5 μL of compounds (final concentration 10 μM) were added to the well and incubated at room temperature for 30 min. 5 μL of Anti-His-Tb cryptate was added at a final concentration of 0.73 nM. After that, streptavidin-XL665 was added to the well at a final concentration of 2.57 nM. The mix was incubated at room temperature for 1 h and HTRF signals were measured at 620 and 665 nm. Results were analyzed with a two-wavelength signal ratio: (intensity (665 nm)/intensity (620 nm)) × 104. The inhibition rate was calculated as follows: inhibition rate (%) = 1 − [(compound signal) − (min signal)]/[(max signal) − (min signal)] × 100, where “max signal” is the signal ratio with the compound vehicle alone (DMSO), and ‘min signal’ is the signal ratio without streptavidin-XL665. Results were expressed as the mean value of triplicate determinations and the positive control was JQ1.

## Figures and Tables

**Table 1:** NMR spectroscopic data for compounds **1** and **2**.

Position	Compound 1 ^a^			Compound 2 ^a^		
	δ_C_, Type	δ_H_ (*J* in Hz)	HMBC ^b^	δ_C_, Type	δ_H_ (*J* in Hz)	HMBC ^b^
1	132.8, CH	7.97, 1H, d (8.8)	3, 5, 19	133.7, CH	9.21, 1H, d (9.2)	3, 5, 19
2	117.5, CH	6.98, 1H, d (8.8)	3, 4, 20	118.9, CH	7.47, 1H, d (9.2)	4, 20
3	160.4, C			166.1, C		
4	113.6, C			111.1, C		
5	140.8, C			124.3, C		
6	206.4, C			187.9, C		
7a	33.6, CH_2_	3.06, 1H, td (17.5, 4.2)	6	126.4, CH	7.04, 1H, (10.0)	4, 9
7b		2.54, 1H, dt (17.5, 3.0)	6, 9			
8a	34.9, CH_2_	2.83, 1H, dt (14.4, 3.0)	5, 6, 7, 9, 10	140.4, CH	8.79, 1H, (10.0)	5, 6, 10
8b		2.27, 1H, td (14.4, 4.2)	7			
9	67.8, C			122.1, C		
10	47.7, CH	2.69, 1H, d (9.3)	5, 9, 11, 14, 15	126.3, C		
11	62.6, CH	4.76, 1H, ddd (9.3, 5.0, 2.9)	10, 12	68.6, CH	5.33, 1H, d (8.5)	12
12	68.4, CH	4.41, 1H, t (3.0)	10, 11, 13, 14	73.0, CH	3.92, 1H, ddd (8.5, 5.4, 3.3)	11
13	65.9, CH	5.03, 1H, d (3.0)	11, 12, 14, 15, 16	66.3, CH	5.65, 1H, dd, (5.3, 3.3)	12
14	121.8, C			118.8, C		
15	132.4, C			141.8, C		
16	156.1, C			154.8, C		
17	113.4, CH	6.80, 1H, d (8.5)	14, 16, 19	121.8, CH	7.43, 1H, d (9.2)	14, 19
18	124.1, CH	7.49, 1H, d (8.5)	15, 16, 20	123.7, CH	8.81, 1H, d (9.2)	15, 16, 20
19	123.5, C			125.7, C		
20	125.0, C			121.4, C		
3-OH		12.68, 1H, s	2, 3, 4		15.23, 1H, s	2, 3, 4
9-OH		5.01, 1H, s				
12-OH					5.51, 1H, d (5.4)	
11-OH		5.12, 1H, d (5.0)				
13-OH		5.72, 1H, s			5.72, 1H, d (5.3)	
16-OH		9.86, 1H, s			10.54, 1H, s	

^a^ Recorded at 600 and 150 MHz in DMSO-*d*_6_; ^b^ HMBC correlations are from proton(s) stated to the indicated carbon.

**Table 2:** NMR spectroscopic data for compound **3**.

Position	Compound 3 ^a^		
	*δ*_C_, Type	*δ*_H_ (*J* in Hz)	HMBC ^b^
1	172.3, C		
2	109.1, C		
3	136.3, C		
4	107.8, CH	5.95, 1H, s	2, 6, 7, 1′
5	160.5, C		
6	100.2, CH	6.35, 1H, s	2, 4, 7
7	162.0, C		
1′	141.0, C		
2′	164.9, C		
3′a	40.5, CH_2_	2.90, 1H, dd (17.1, 6.5)	1′, 4′, 5′, 2′-CH_3_
3′b		2.41, 1H, d (17.1)	1′, 4′
4′	71.4, CH	4.21, 1H, br s	2′, 5′
5′	205.9, C		
7-OCH_3_	55.2, CH_3_	3.74, 3H, s	1, 6, 7
2′-CH_3_	17.4, CH_3_	1.90, 3H, s	2, 4, 1′, 2′, 3′, 5′

^a^ Recorded at 400 and 100 MHz in DMSO-*d*_6_; ^b^ HMBC correlations are from proton(s) stated to the indicated carbon.

**Table 3:** NMR spectroscopic data for compound **4**.

Position	Compound 4 ^a^		
	*δ*_C_, Type	*δ*_H_ (*J* in Hz)	HMBC ^b^
1	172.1, C		
2	128.0, C		
3	109.5, CH	6.40, 1H, s	4, 5, 6, 8
4	168.7, C		
1′	144.3, CH	9.07, 1H, s	1, 4, 5
2′-NH		9.25, 1H, s	1, 3
3′	169.4, C		
4′	59.4, CH_2_	4.32, 2H, s	6, 7
3′-CH_3_	23.3, CH_3_	2.10, 3H, s	1
4′-OH		5.73, 1H, s	

^a^ Recorded at 400 and 100 MHz in DMSO-*d*_6_; ^b^ HMBC correlations are from proton(s) stated to the indicated carbon.

**Table 4:** NMR spectroscopic data for compound **5**.

Position	Compound 5 ^a^		
	*δ*_C_, Type	*δ*_H_ (*J* in Hz)	HMBC ^b^
1a	69.6, CH_2_	4.04, 1H, m	
1b		3.85, 1H, m	
2	53.5, CH	4.04, 1H, m	
3	72.4, CH	4.10, 1H, m	
4	128.6, CH	5.45, 1H, dd (15.8, 6.0)	
5	134.6, CH	5.72, 1H, d (15.8)	
6	27.9, CH_2_	2.02, 2H, m	
7	123.3, CH	5.08, 1H, t (6.0)	6
8	136.1, C		
9	40.0, CH_2_	1.95, 2H, t (7.5)	6, 8, 10, 18
10	28.3, CH_2_	1.36, 2H, m	
11	29.4, CH_2_	1.26, 2H, m	
12–14	29.6–30.0, CH_2_	1.26 × 3, 6H, m	
15	32.1, CH_2_	1.26, 2H, m	16, 17
16	22.8, CH_2_	1.26, 2H, m	15, 17
17	14.3, CH_3_	0.89, 3H, t (6.8)	15, 16
18	16.2, CH_3_	1.57, 3H, s	6, 8, 9
1′	174.8, C		
2′	73.1, CH	4.56, 1H, d (5.3)	
3′	126.6, CH	5.49, 1H, dd (15.8, 4.2)	
4′	134.3, CH	5.85, 1H, dt (15.8, 5.8)	2′
5′	32.9, CH_2_	2.00, 2H, m	6′
6′	32.7, CH_2_	1.35, 2H, m	
7′–12′	29.6–30.0, CH_2_	1.26 × 6, 12H, m	
13′	29.4, CH_2_	1.26, 2H, m	
14′–16′	29.6–30.0, CH_2_	1.26 × 3, 6H, m	
17′	32.1, CH_2_	1.26, 2H, m	18′, 19′
18′	22.8, CH_2_	1.26, 2H, m	17′, 19′
19′	14.3, CH_3_	0.89, 3H, t (6.8)	17′, 18′
1″	103.1, CH	4.37, 1H, s	
2″	73.4, CH	3.35, 1H, m	
3″	76.1, CH	3.36, 1H, m	
4″	69.6, CH	3.52, 1H, m	
5″	76.1, CH	3.53, 1H, m	
6″	61.1, CH_2_	3.85, 2H, m	
NH		7.39, 1H, s	

^a^ Recorded at 400 and 100MHz in CDCl_3_; ^b^ HMBC correlations are from proton(s) stated to the indicated carbon.

**Table 5:** Inhibition rates of compounds **1**–**24** against BRD4 protein ^a^.

Compounds	Inhibition Rate	
	10 µM	IC_50_
**1**	57.7%	
**2**	88.1%	
**3–24**	<35.0%	
**JQ1** ^b^		239.6 nM

^a^ Results are expressed as means ± SD (*n* = 3); ^b^ Positive control substance.

## Supplementary Materials

The following are available online at www.mdpi.com/1660-3397/15/3/76/s1, Figures S1–S38: HR-ESI-MS, UV, 1D, and 2D NMR spectra of compounds **1**–**5**, Table S1: ^1^H and ^13^C NMR data of compound **6** (MeOH-*d_4_*), Table S2: abbreviations used in the article.
