# Supplementary material for: Inhibitors of BRD4 Protein from a Marine-Derived Fungus Alternaria sp. NH-F6
Source: Mar Drugs. 2017 Mar 16;15(3):76. doi: 10.3390/md15030076 (PMC5367033; doi:10.3390/md15030076)
Supplement: Supplementary file 2 [file marinedrugs-15-00076-s002.pdf]

# SupplementaryMaterials: Inhibitors of BRD4 Protein from a Marine-derived Fungus *Alternaria* sp. NH-F6

**Hui Ding, Dashan Zhang, Biao Zhou and Zhongjun Ma***

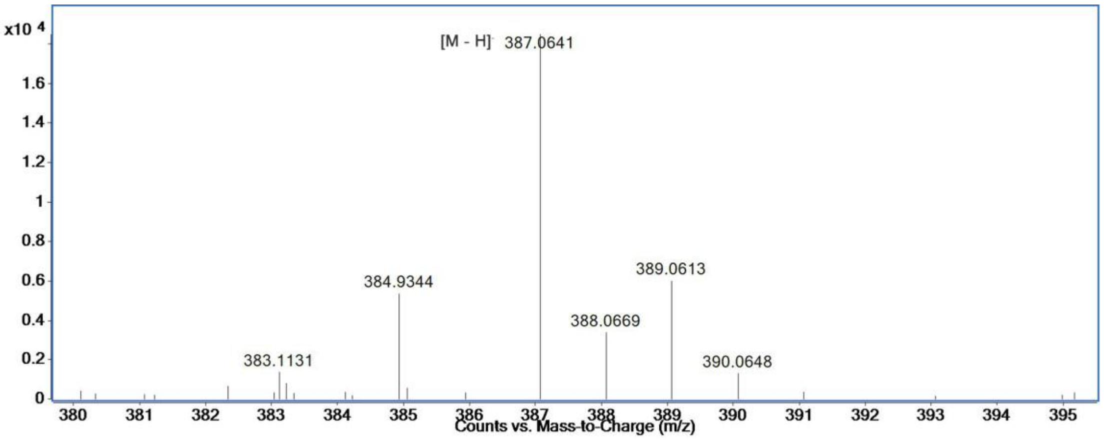


**Figure S1.** HR-ESI-MS spectrum of the new compound **1**

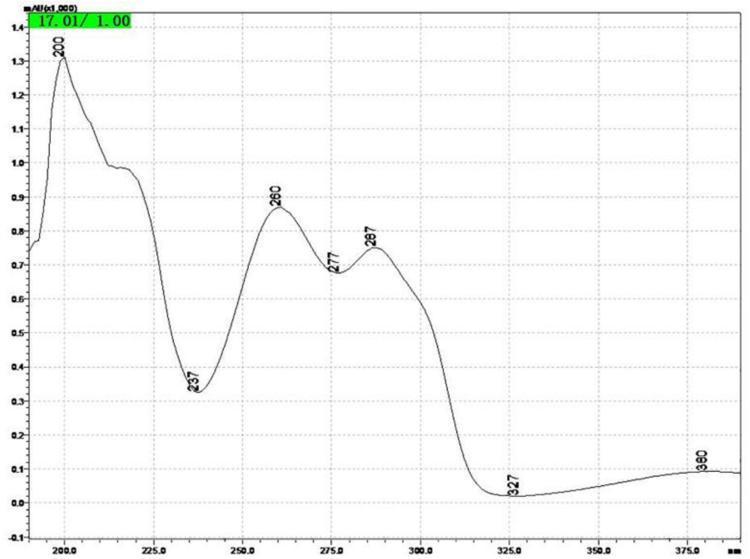


**Figure S2.** UV spectrum of the new compound **1**

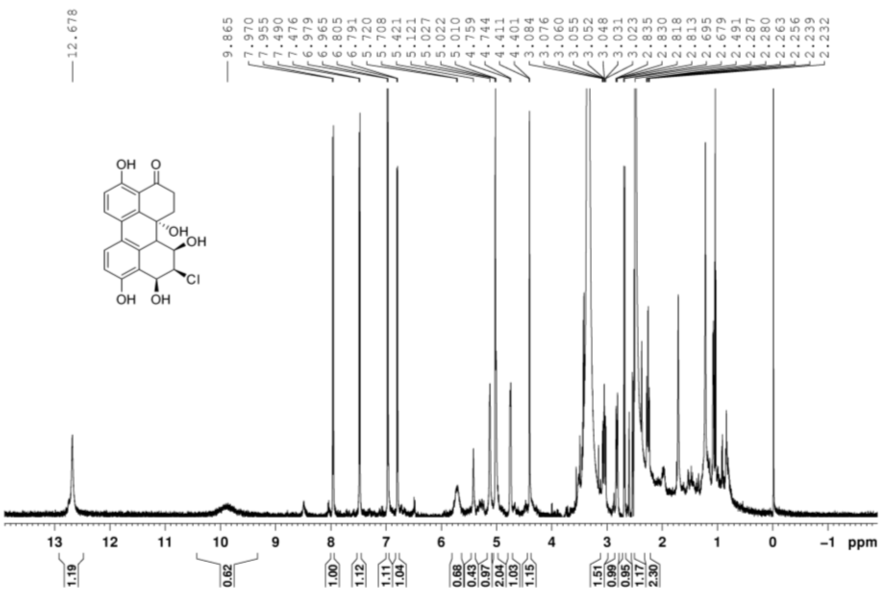

**Figure S3.** $^{1}$H NMR (600 MHz, DMSO-$d_6$) spectrum of the new compound **1**

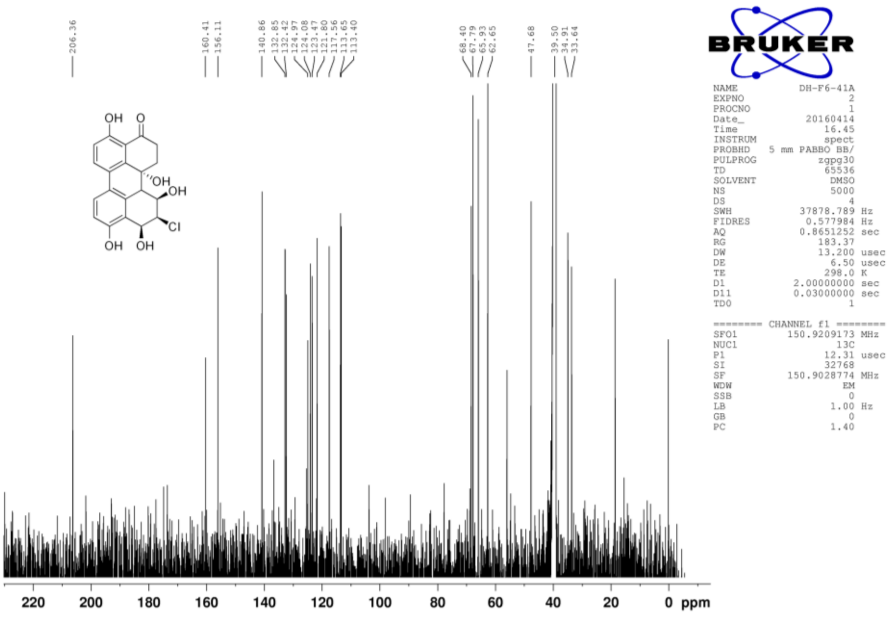

**Figure S4.** $^{13}$C NMR (150 MHz, DMSO-$d_6$) spectrum of the new compound **1**

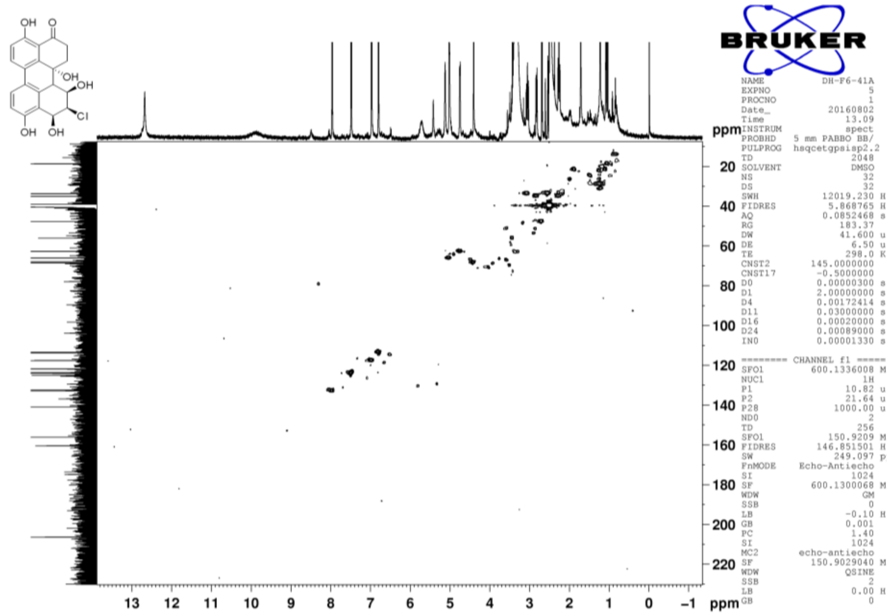


**Figure S5.** HSQC spectrum of the new compound **1**

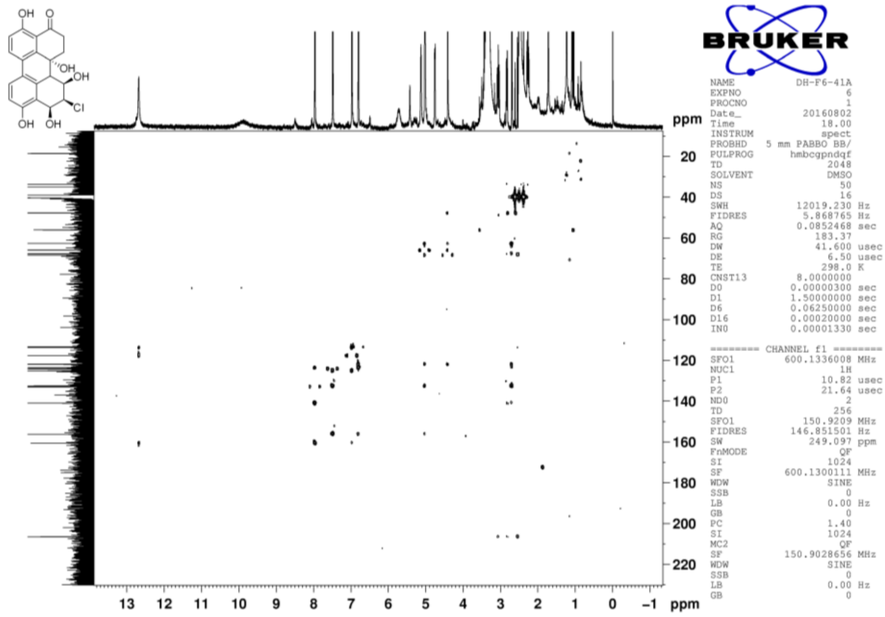


**Figure S6.** HMBC spectrum of the new compound **1**

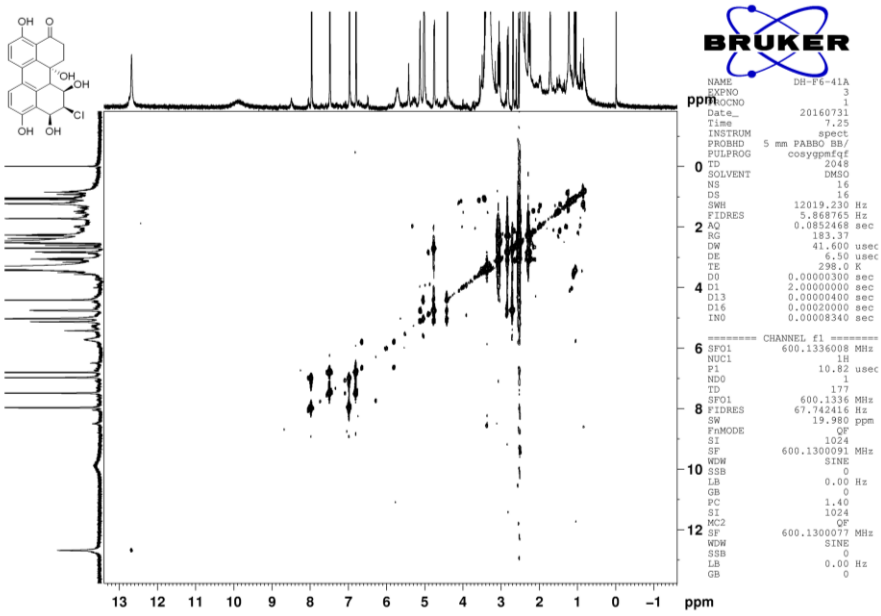


**Figure S7.** $^{1}H$-$^{1}H$ COSY spectrum of the new compound **1**

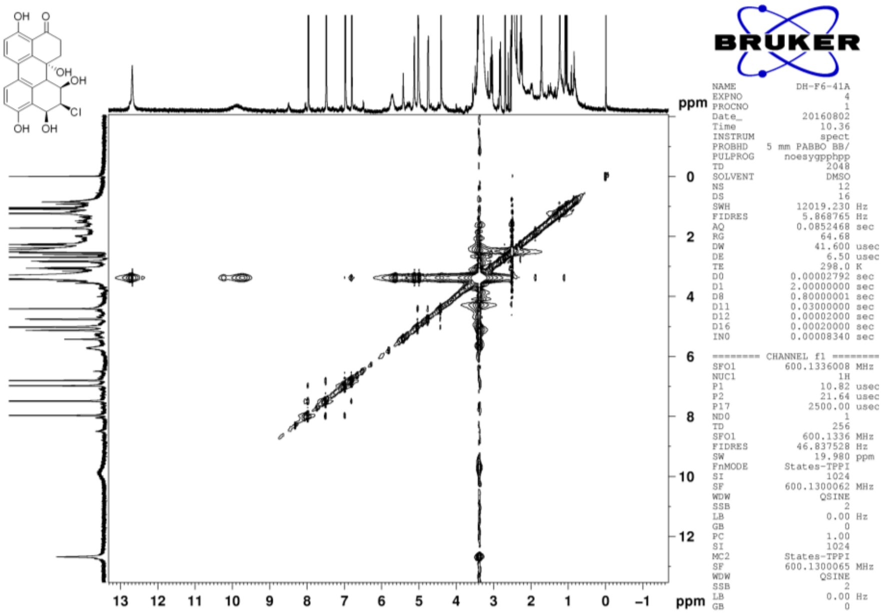


**Figure S8.** NOESY spectrum of the new compound **1**

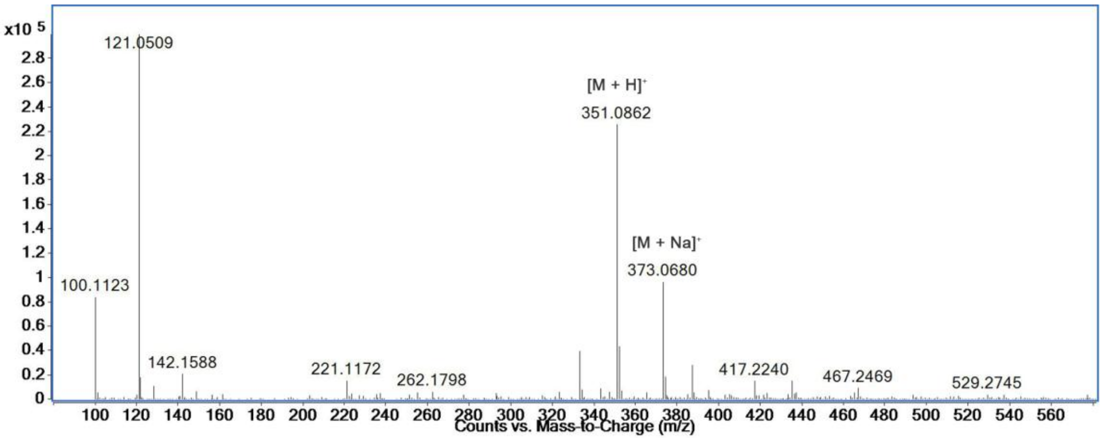


**Figure S9.** HR-ESI-MS spectrum of the new compound **2**

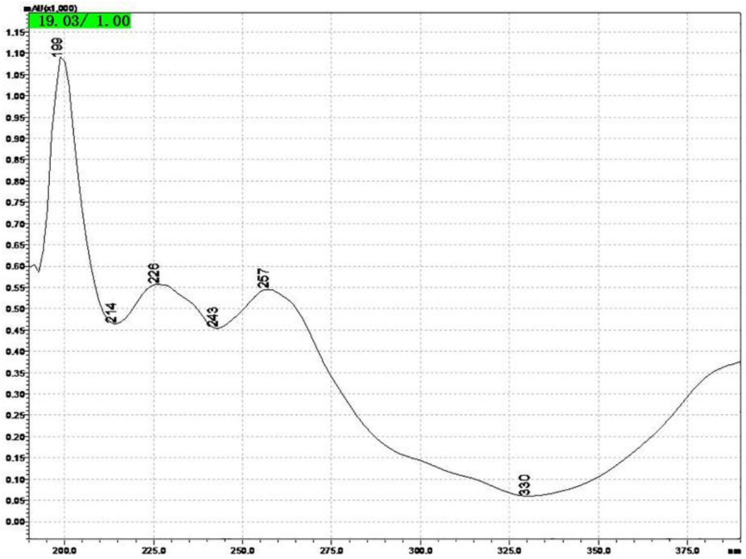


**Figure S10.** UV spectrum of the new compound **2**

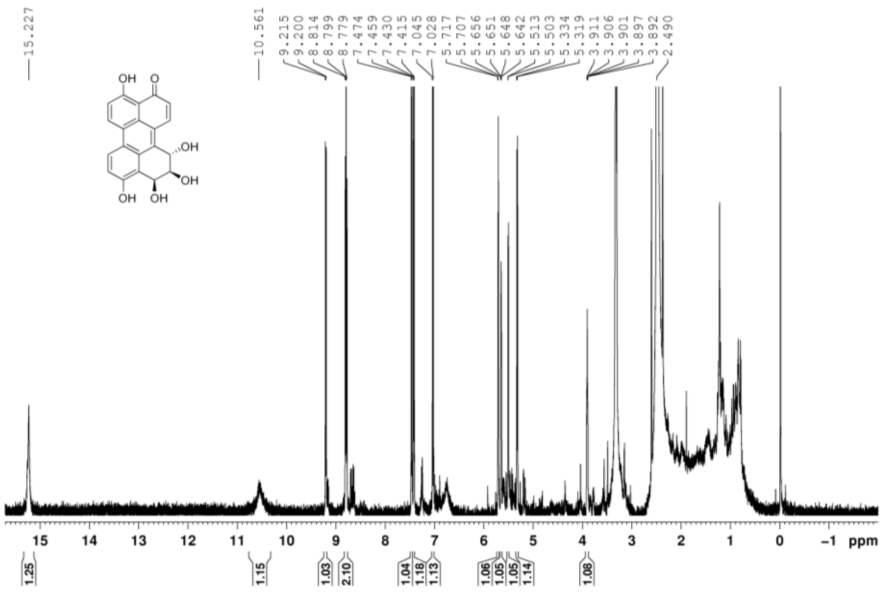


**Figure S11.** $^{1}$H NMR (600 MHz, DMSO-*d*$_6$) spectrum of the new compound **2**

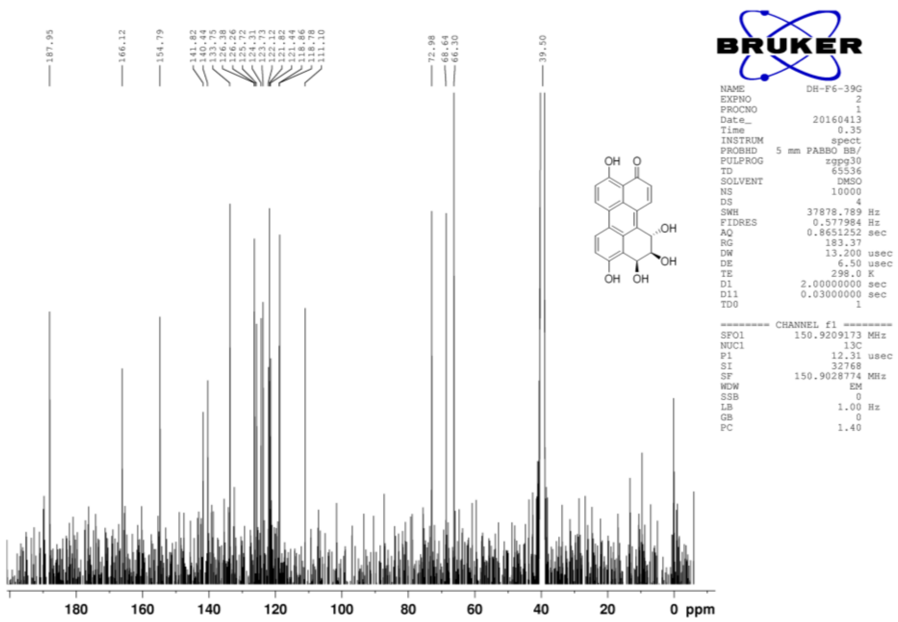


**Figure S12.** $^{13}$C NMR (150 MHz, DMSO-*d*$_6$) spectrum of the new compound **2**

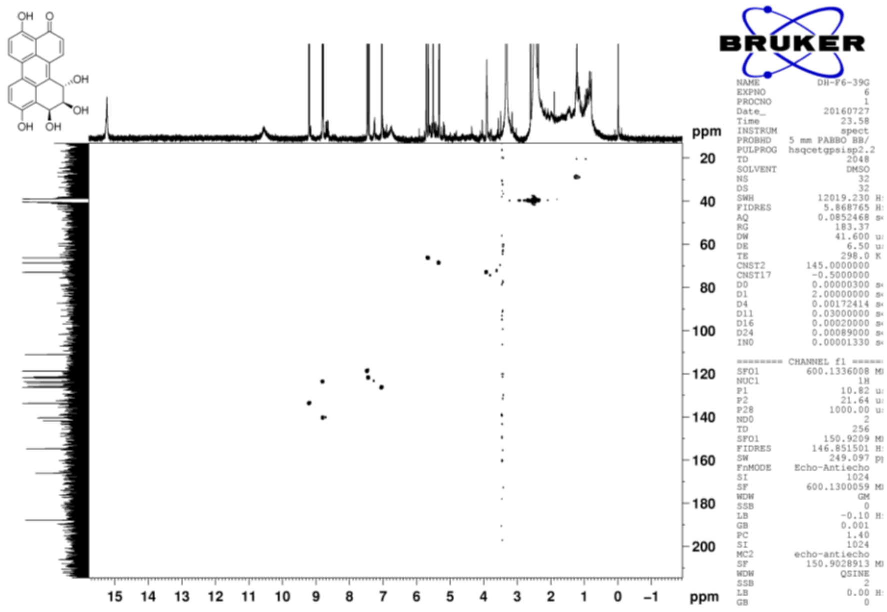


**Figure S13.** HSQC spectrum of the new compound **2**

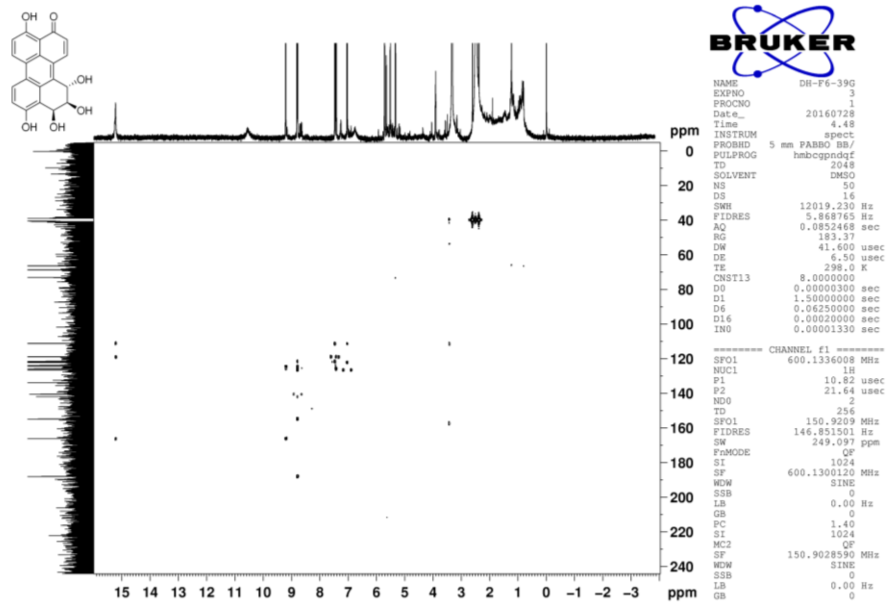


**Figure S14.** HMBC spectrum of the new compound **2**

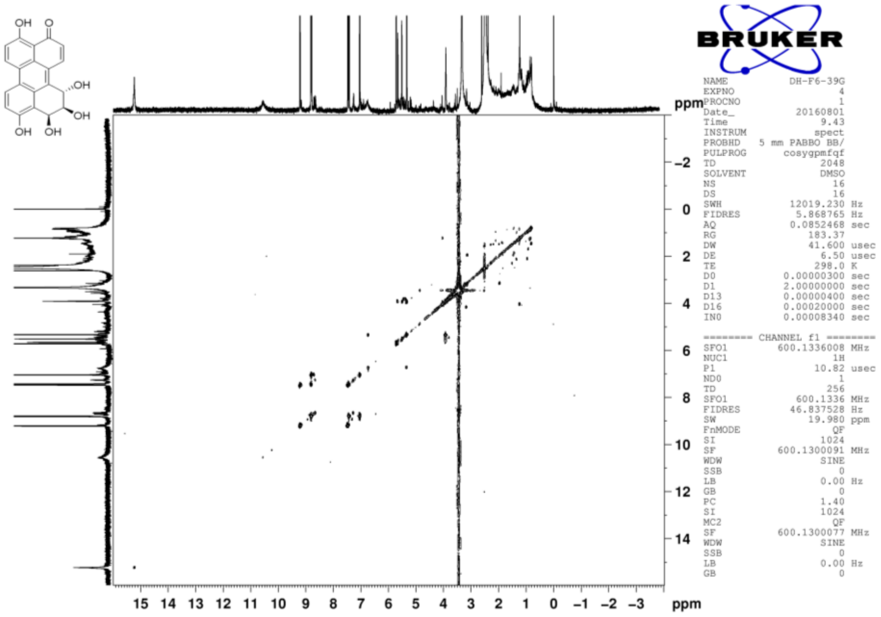


**Figure S15.** $^1$H-$^1$H COSY spectrum of the new compound **2**

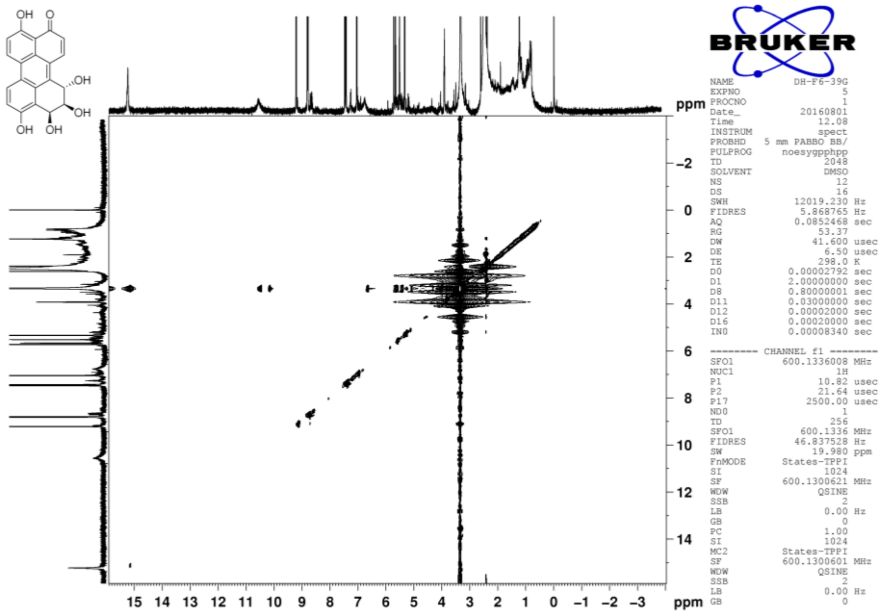


**Figure S16.** NOESY spectrum of the new compound **2**

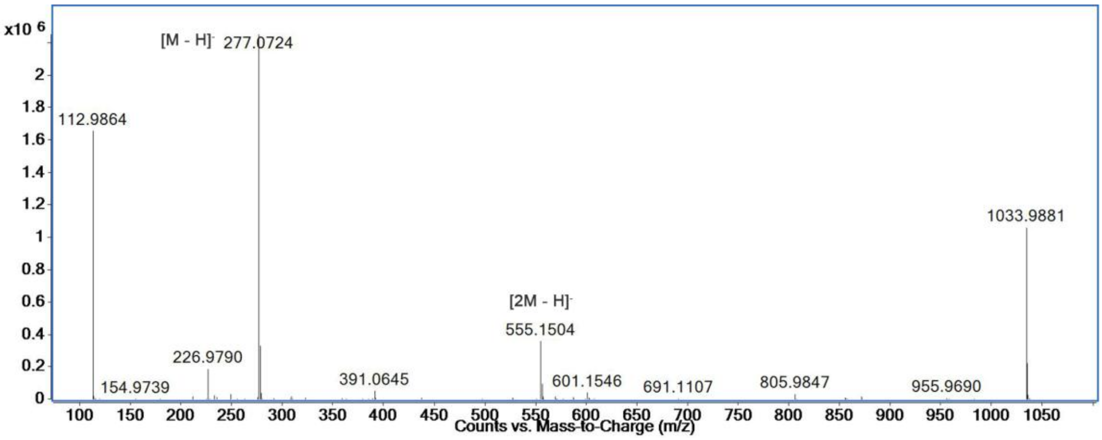


**Figure S17.** HR-ESI-MS spectrum of the new compound **3**

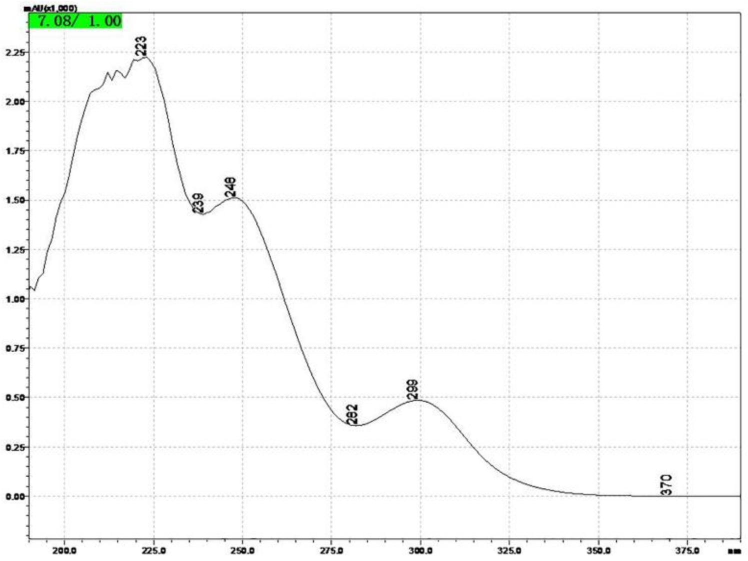


**Figure S18.** UV spectrum of the new compound **3**

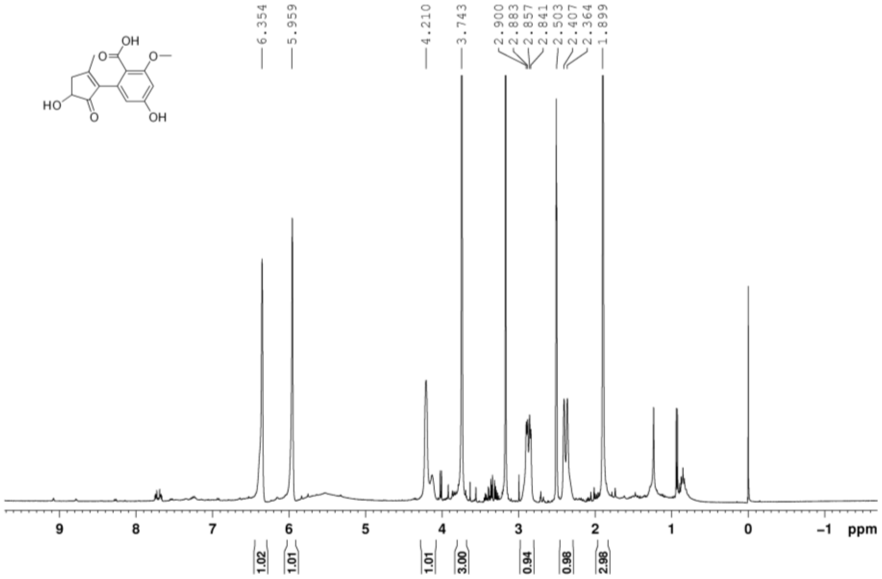


**Figure S19.** $^{1}$H NMR (400 MHz, DMSO-$d_6$) spectrum of the new compound **3**

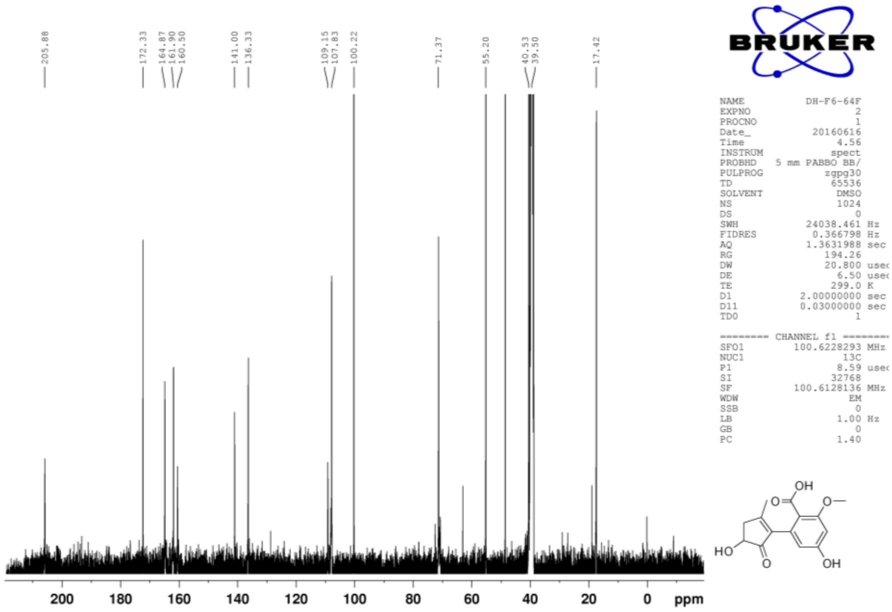


**Figure S20.** $^{13}$C NMR (100 MHz, DMSO-$d_6$) spectrum of the new compound **3**

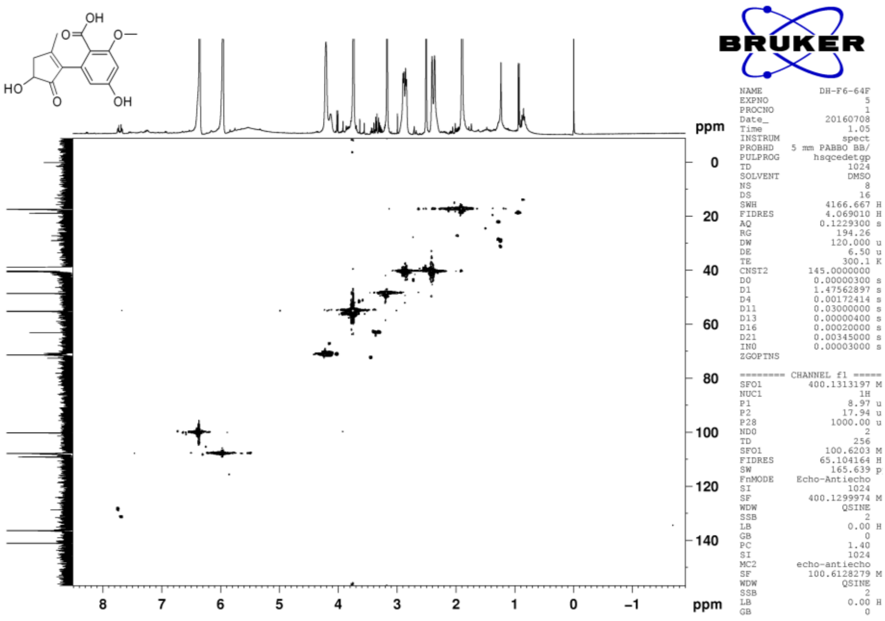


**Figure S21.** HSQC spectrum of the new compound **3**

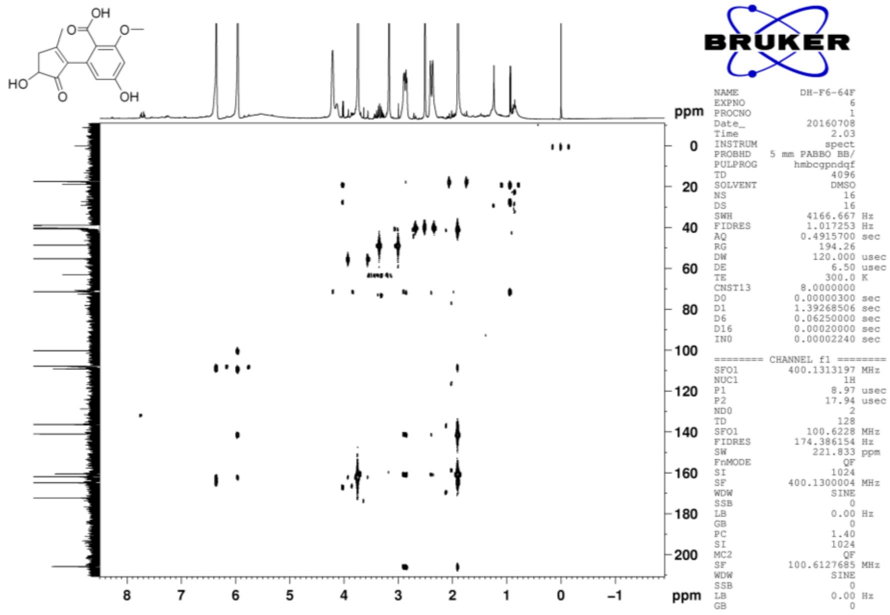


**Figure S22.** HMBC spectrum of the new compound **3**

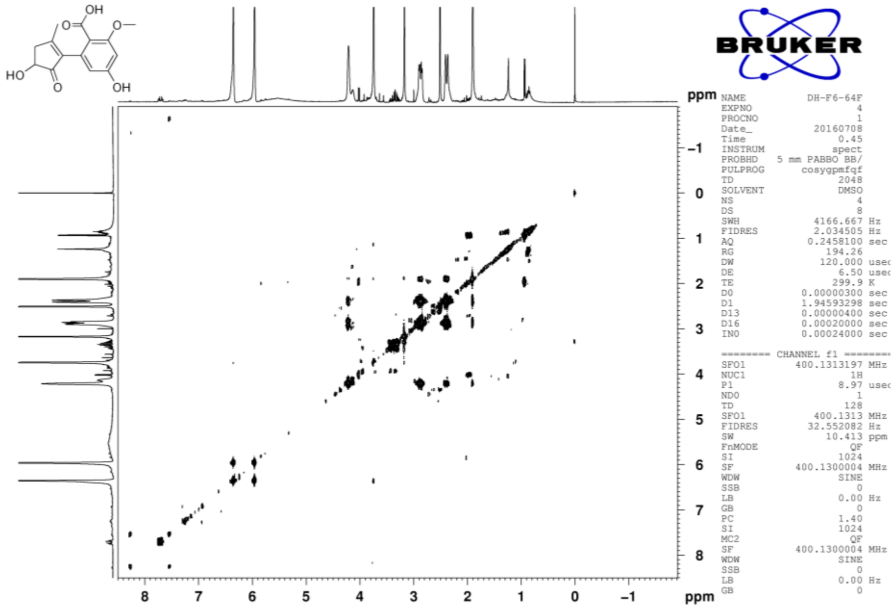


**Figure S23.** $^{1}H$-$^{1}H$ COSY spectrum of the new compound **3**

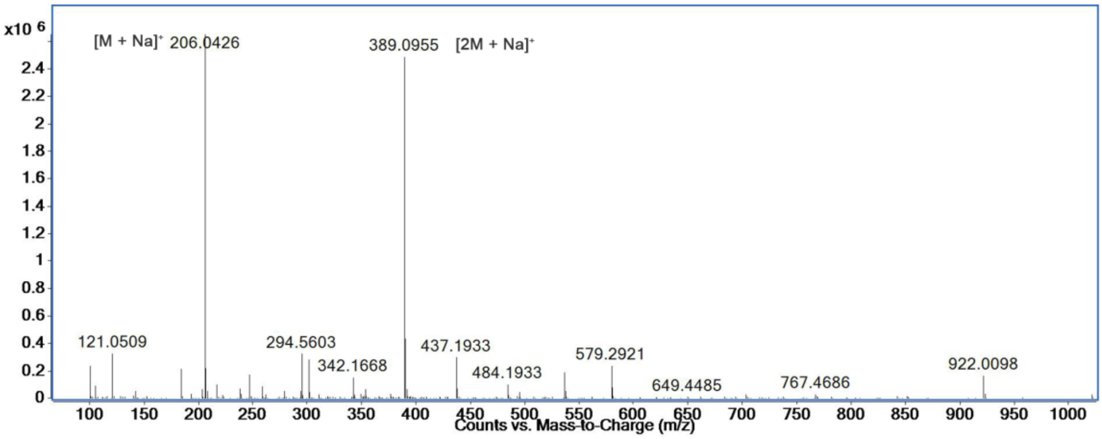


**Figure S24.** HR-ESI-MS spectrum of the new compound **4**

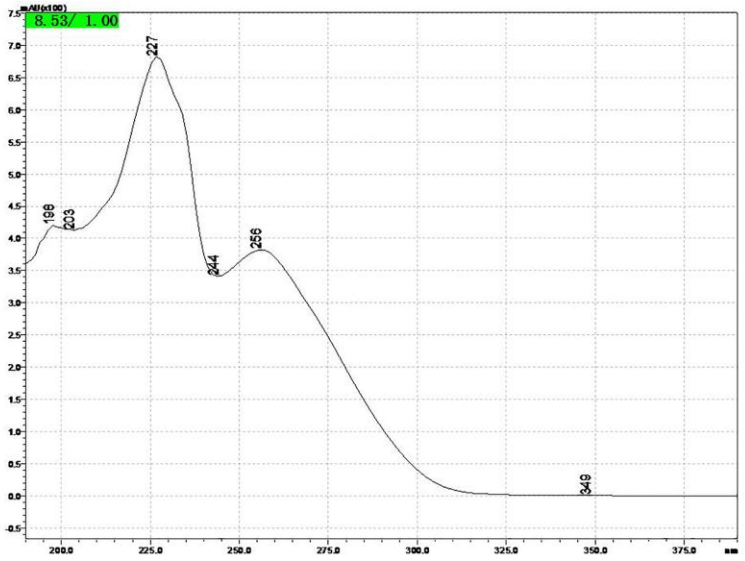

**Figure S25.** UV spectrum of the new compound **4**

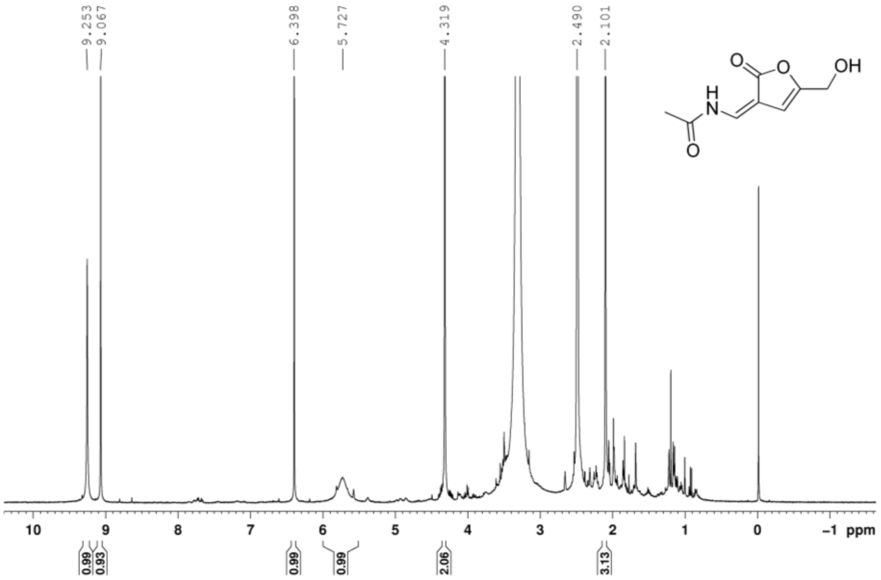

**Figure S26.** $^{1}$H NMR (400 MHz, DMSO-$d_6$) spectrum of the new compound **4**

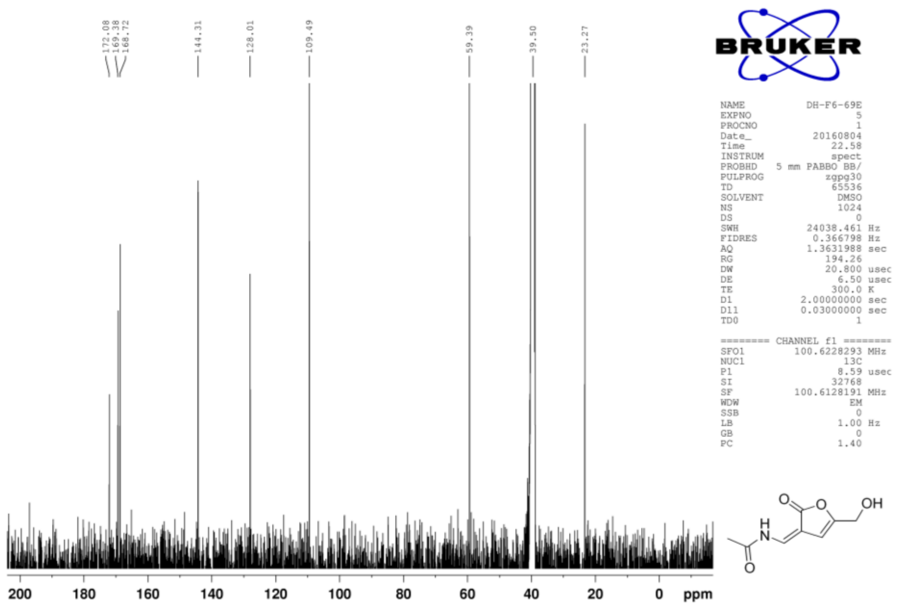


**Figure S27.** $^{13}$C NMR (100 MHz, DMSO-$d_6$) spectrum of the new compound **4**

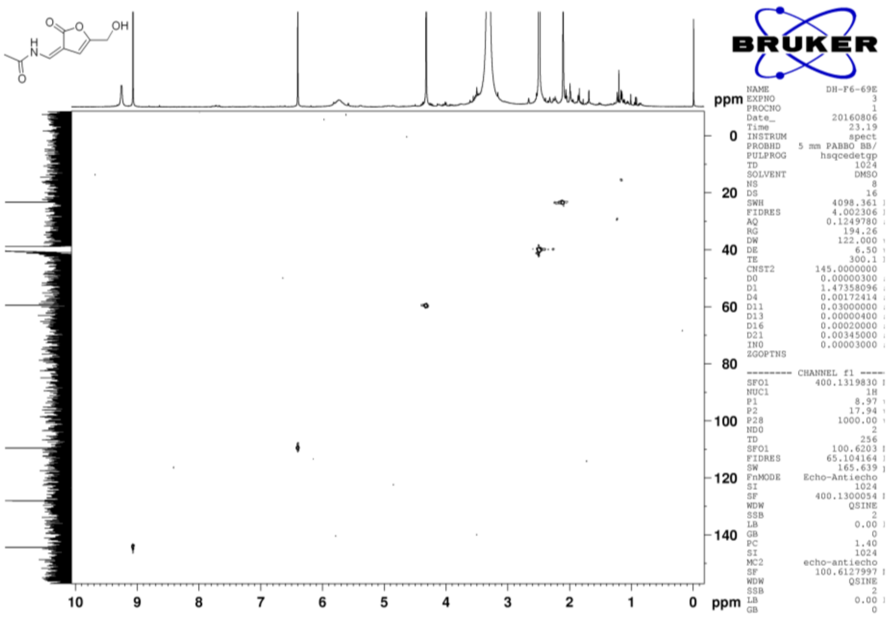


**Figure S28.** HSQC spectrum of the new compound **4**

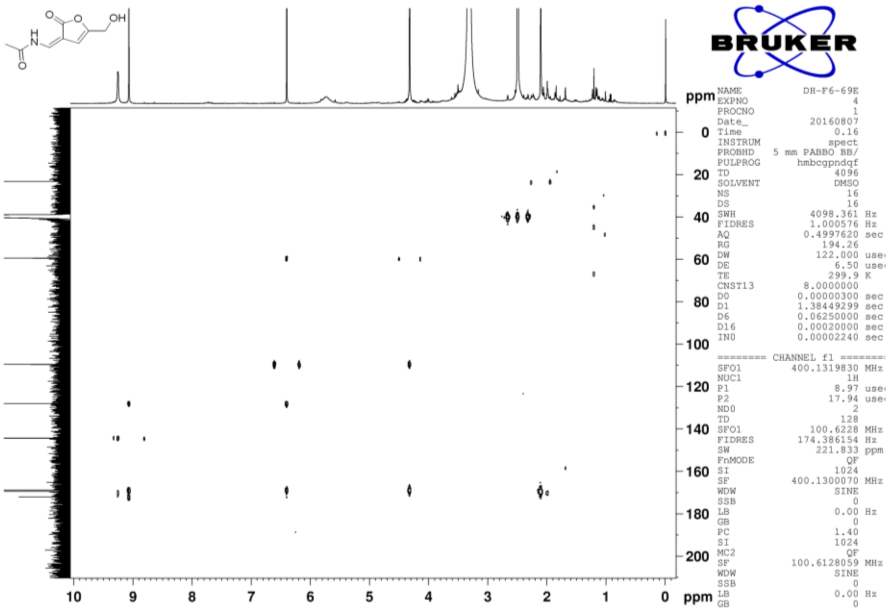


**Figure S29.** HMBC spectrum of the new compound **4**

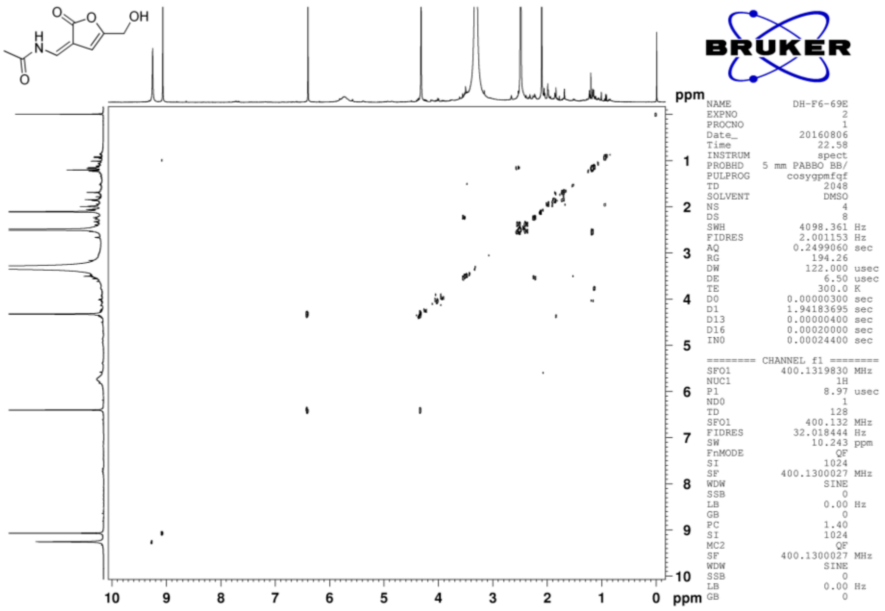


**Figure S30.** $^{1}H$-$^{1}H$ COSY spectrum of the new compound **4**

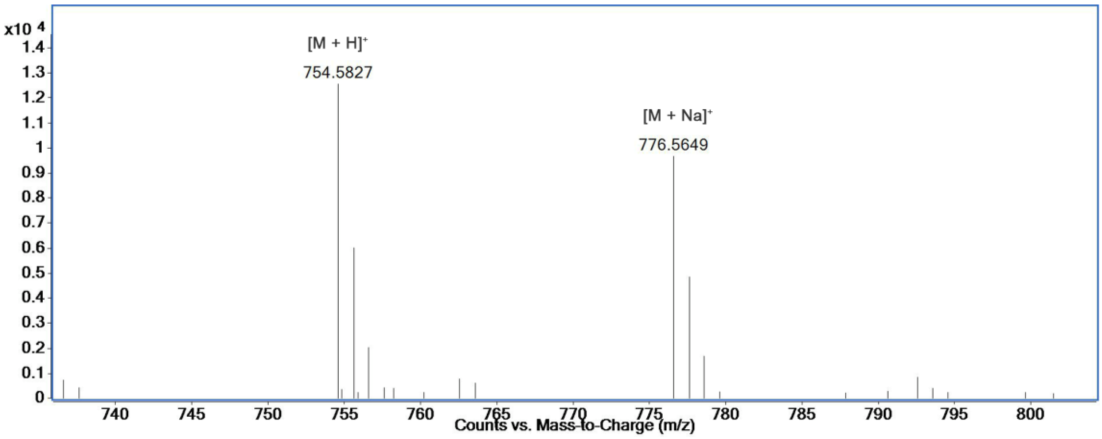


**Figure S31.** HR-ESI-MS spectrum of the new compound **5**

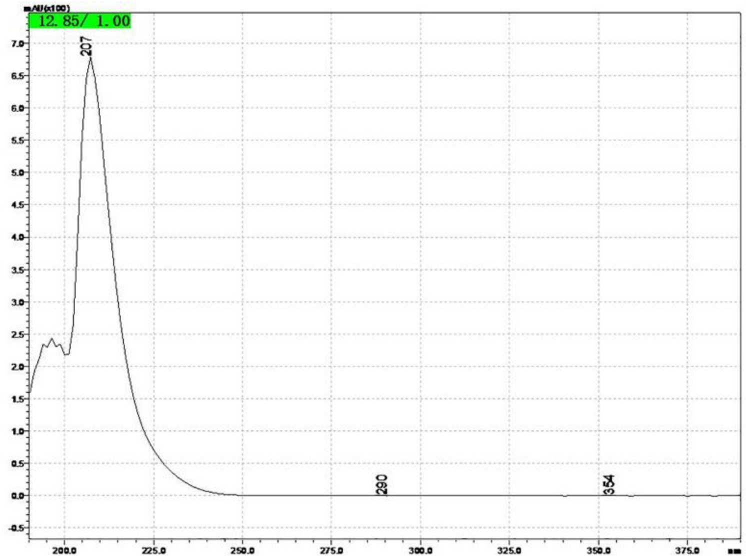


**Figure S32.** UV spectrum of the new compound **5**

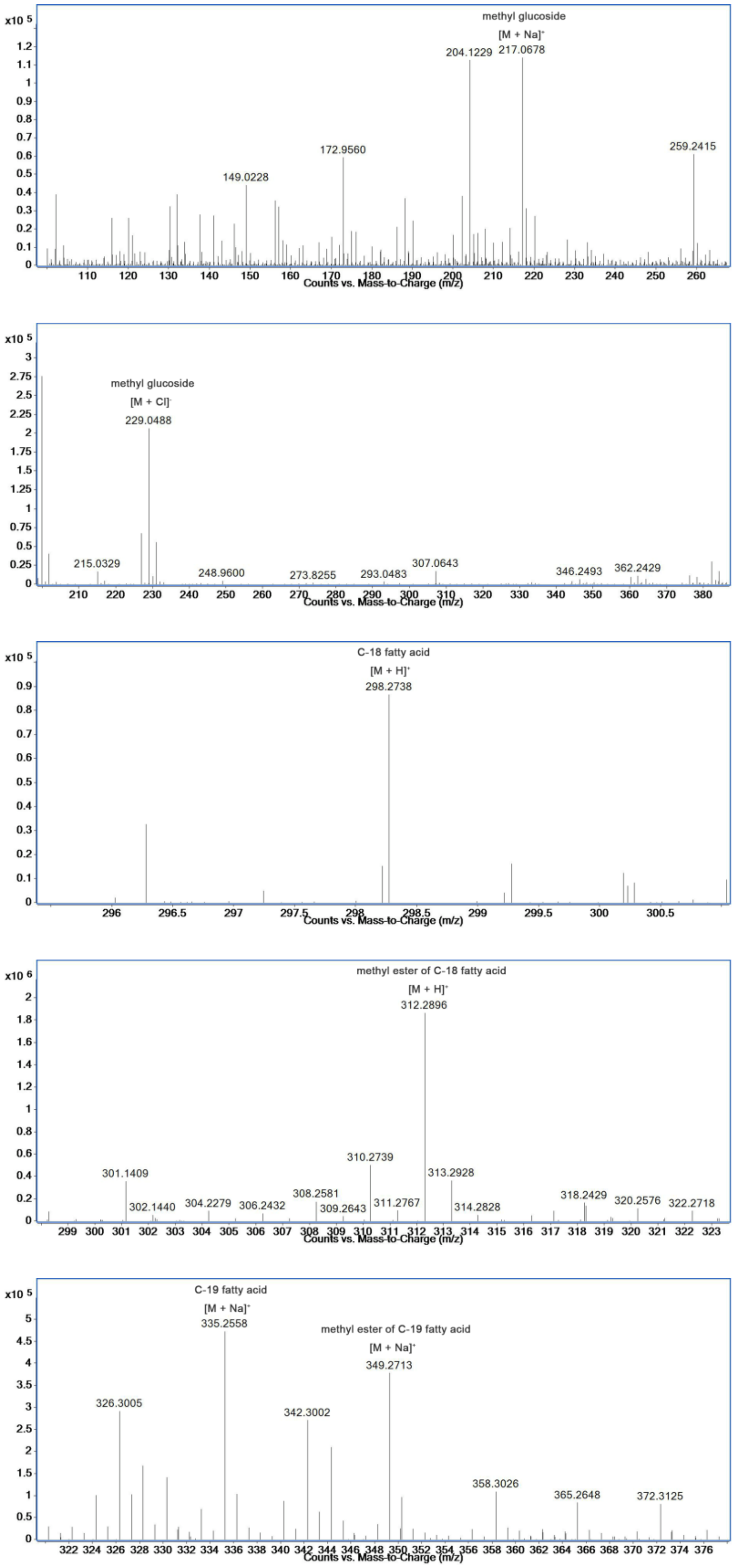


**Figure S33.** The methanolysis products' HR-ESI-MS spectra of the new compound **5**

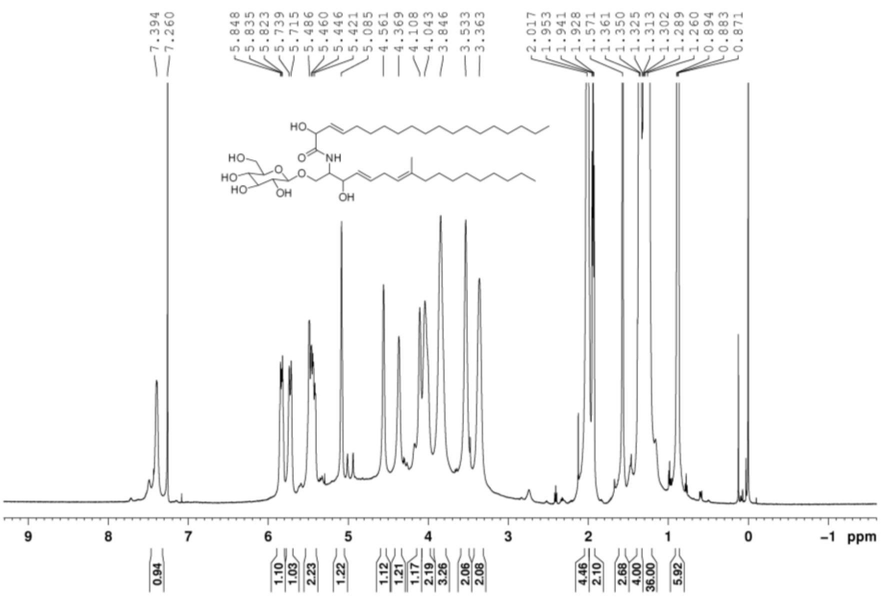


**Figure S34.** $^{1}$H NMR (400 MHz, $CDCl_3$) spectrum of the new compound **5**

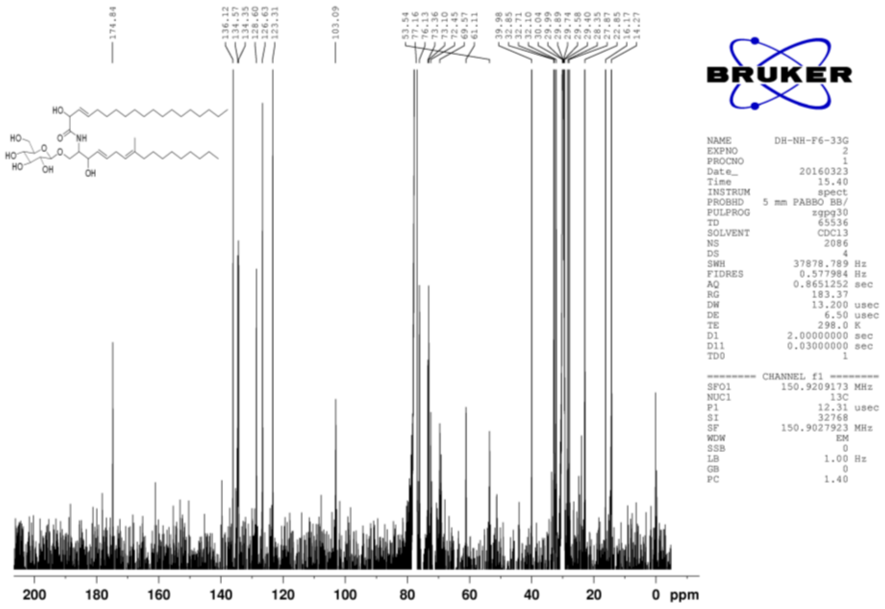


**Figure S35.** $^{13}$C NMR (100 MHz, $CDCl_3$) spectrum of the new compound **5**

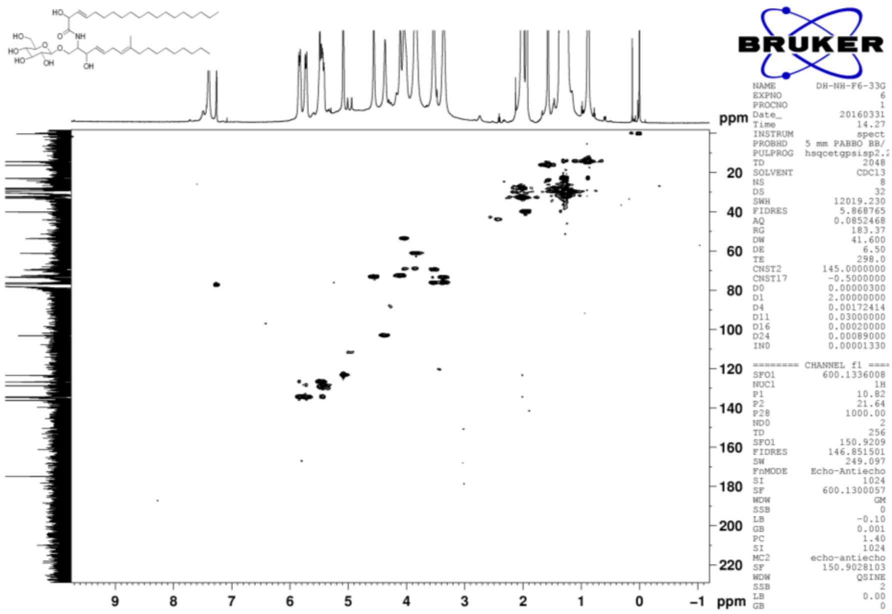


**Figure S36.** HSQC spectrum of the new compound **5**

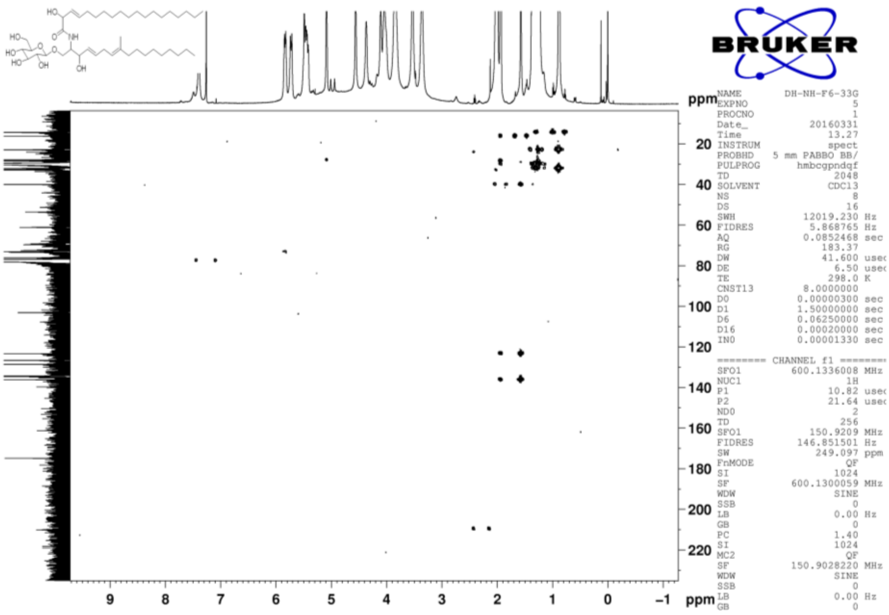


**Figure S37.** HMBC spectrum of the new compound **5**

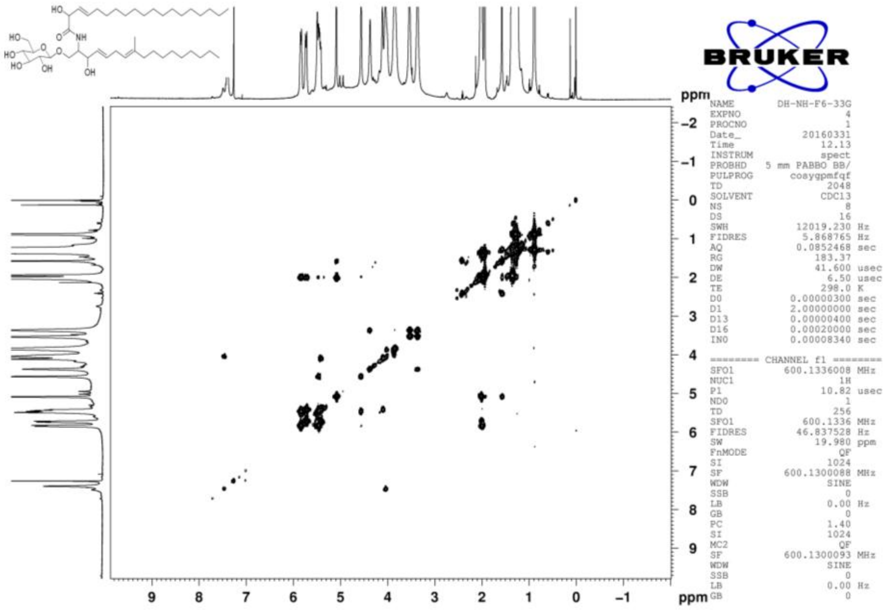


**Figure S38.** $^{1}$H-$^{1}$H COSY spectrum of the new compound **5**

**Table S1.** $^{1}$H NMR (600 MHz) and $^{13}$C NMR (150 MHz) data of compound **6** in MeOH-*d*$_4$.

| position | compound **6** | |
|---|---|---|
| | $\delta_C$, type | $\delta_H$ (*J* in Hz) |
| 1 | 165.4, C | |
| 2 | 109.3, C | |
| 3 | 137.1, C | |
| 4 | 109.9, CH | 6.07, 1H, d (2.3) |
| 5 | 163.8, CH | 6.40, 1H, d (2.3) |
| 6 | 101.4, C | |
| 7 | 168.5, C | |
| 1' | 142.6, C | |
| 2' | 165.3, C | |
| 3'a | 41.8, $CH_2$ | 3.04, 1H, dd (17.0, 6.5) |
| 3'b | | 2.53, 1H, d (17.0) |
| 4' | 73.4, CH | 4.33, 1H, dd (6.5, 2.8) |
| 5' | 209.7, C | |
| 5-$OCH_3$ | 55.8, $CH_3$ | 3.78, 3H, s |
| 2'-$CH_3$ | 17.9, $CH_3$ | 1.99, 3H, s |

**Table S2.** Abbreviations

| full name | abbreviation |
|---|---|
| nuclear magnetic resonance | NMR |
| high resolution electrospray ionization mass spectrometry | HR-ESI-MS |
| bromodomain-containing protein 4 | BRD4 |
| human immunodeficiency virus | HIV |
| bromodomain | BRD |
| bromodomain and extra-terminal domain | BET |
| heteronuclear singular quantum correlation | HSQC |
| heteronuclear multiple bond correlation | HMBC |
| homonuclear chemical shift correlation spectroscopy | $^1H$-$^1H$ COSY |
| nuclear overhauser effect spectroscopy | NOESY |
| infrared ray | IR |
| high performance liquid chromatography | HPLC |
| medium performance liquid chromatography | MPLC |
| time-resolved fluorescence resonance energy transfer | TR-FRET |
| bromodomain 1 | BD1 |
| ribosomal deoxyribose nucleic acid | rDNA |
| potato dextrose agar | PDA |
| potato dextrose broth | PDB |
| *chemical formula | $C_{20}H_{17}ClO_6$, $C_{20}H_{14}O_6$, et al. |

*1. Confirm whether a heteroatom is in the structure according to the isotopic peak in HR-ESI-MS spectrum. For example, if the isotopic peak is 3:1, there is a chlorine atoms in the structure.

2. Count the number of carbon and hydrogen atoms in NMR spectrum.

3. Calculate the sum of all atoms' atomic mass using ChemDraw software.

molecular formula (calculated) = (carbon atom's atomic mass) × (the number of carbon atoms) + (hydrogen atom's atomic mass) × (the number of hydrogen atoms) + (oxygen atom's atomic mass) × (the number of oxygen atoms) + (nitrogen atom's atomic mass) × (the number of nitrogen atoms) + (chlorine atom's atomic mass) × (the number of chlorine atoms)

Atomic mass (calculated): C, 12.0000, H, 1.0078, O, 15.9949, N, 14.0031, Cl, 34.9689

4. Compare the calculated value with the result of HR-ESI-MS until they are almost the same (± 0.0006).
